# Stereoselective total synthesis and structural revision of the diacetylenic diol natural products strongylodiols H and I

**DOI:** 10.3762/bjoc.14.206

**Published:** 2018-09-04

**Authors:** Pamarthi Gangadhar, Sayini Ramakrishna, Ponneri Venkateswarlu, Pabbaraja Srihari

**Affiliations:** 1 Department of Organic Synthesis and Process Chemistry, CSIR-Indian Institute of Chemical Technology, Hyderabad-500007, Telangana, India; 2 Department of Chemistry, S. V. U. College of Sciences, Tirupati-517502, India

**Keywords:** alkylation, Cadiot–Chodkiewicz coupling, Corey–Bakshi–Shibata reduction, Mosher’s analysis, Wittig reaction

## Abstract

The stereoselective total synthesis of strongylodiol H and I has been accomplished. The synthetic procedure comprised the stereoselective reduction of a ketone functionality in an ene–yne–one employing CBS as a catalyst and a Cadiot–Chodkiewicz coupling reaction as the key reaction steps. A common aldehyde intermediate has been used for the synthesis of both strongylodiols.

## Introduction

**Figure 1:**
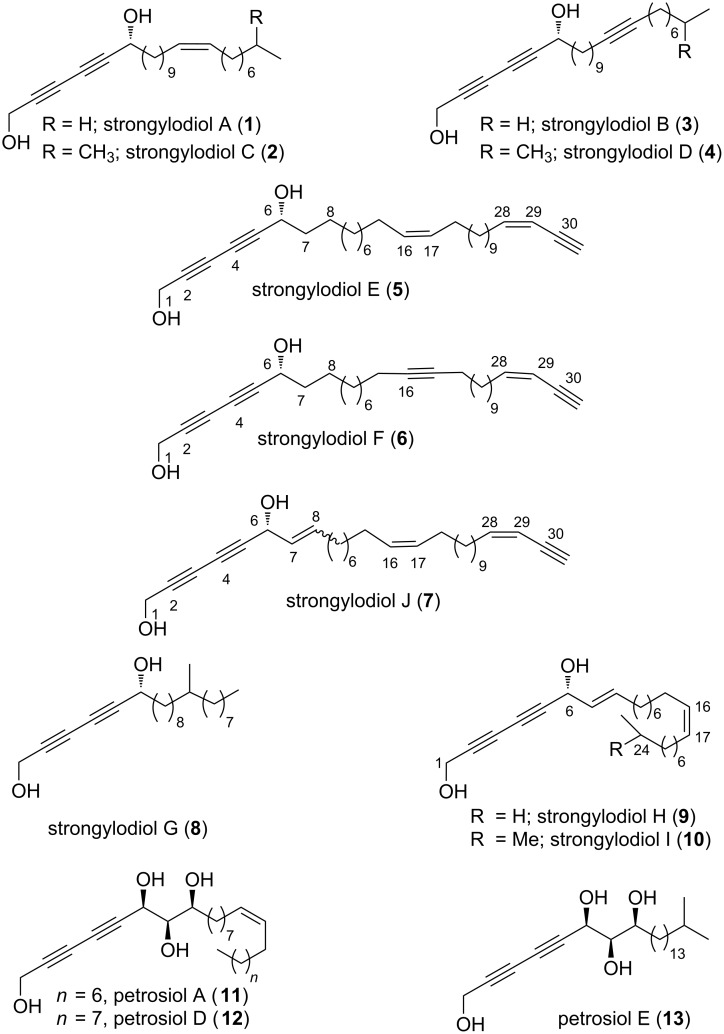
Proposed structures of a selection of diacetylenic polyol natural products.

Diacetylenic polyol compounds [1–2] originated from marine sources continue to attract significant interest owing to their structural architectures and impressive biological properties that include antibacterial [3], anticancer [4–6], antiviral [7] and neuritogenic activities [8]. Watanabe et al. [9–10] have isolated strongylodiols A–J (**1**–**10,** Figure 1) [11] from the Okinawan marine sponge of the genus *Petrosia* (*Strongylophora*). The structural elucidation was accomplished by spectroscopic analyses and chemical reactions. It was found that these strongylodiols were present as an enantiomeric mixture with different ratios after analysis of their corresponding MNA ((*R*)- and (*S*)-methoxy(2-naphthyl)acetic acid) derivatives. Though petrosiols A, D and E along with strongylodiols C and D were found to display neuronal differentiation of PC12 cells in a dose-dependent manner and induce neuronal outgrowth, strongylodiols C and D also displayed cytotoxicity at higher concentrations [11]. There have been few contributions on the total synthesis of strongylodiols [12–13] employing alkynylation of an unsaturated aliphatic aldehyde catalyzed by Trost’s pro-phenol ligand [12,14], β-elimination of epoxy chloride [15], Noyori’s asymmetric reduction of ynones [16], diyne addition to long chain aliphatic aldehydes in the presence of *N*-methylephedrine [17] or an amino alcohol–zinc complex [18] and the Cadiot–Chodkiewicz cross-coupling reaction as key steps [14–16].

In continuation to our research interest on the synthesis of acetylenic compounds [19–21], recently we have accomplished the total synthesis of the diacetylenic polyol natural products petrosiols A, D, E (**11**,**12**,**13**) [22–23] and strongylodiols A–D (**1**–**4**) [24]. Herein we describe the total synthesis of strongylodiols H and I (**9** and **10**).

## Results and Discussion

### Retrosynthesis for strongylodiol H and strongylodiol I

**Scheme 1:**
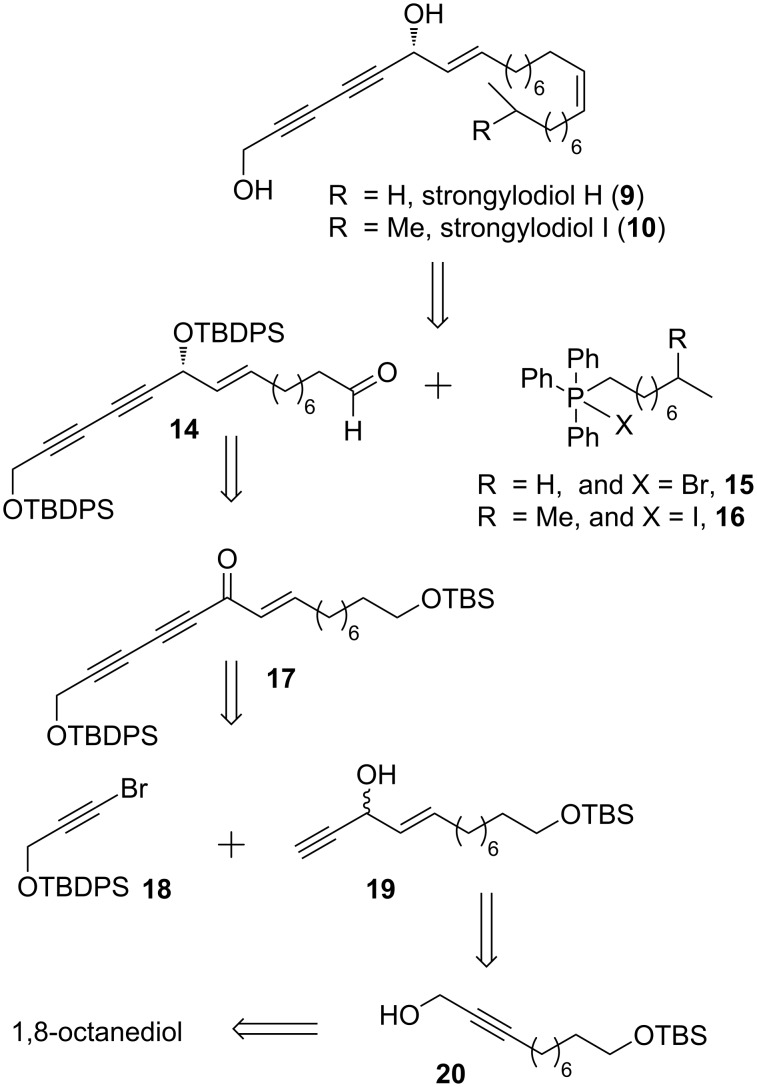
Retrosynthesis of strongylodiols H and I.

The retrosynthetic analysis for strongylodiols H and I is delineated in Scheme 1. We envisaged that the target molecules strongylodiol H (**9**) and strongylodiol I (**10**) can be synthesized by a Wittig reaction of a common intermediate aldehyde **14** with triphenylphosphonium Wittig salts **15** and **16**, respectively, followed by the desilylation (TBDPS removal) to yield the title products. The intermediate aldehyde **14** can be synthesized from ketone **17** in a four-step sequence by a stereoselective keto reduction, TBDPS protection, TBS deprotection and an oxidation reaction. The ketone **17** can be easily synthesized by a coupling reaction of **18** with **19** followed by an oxidation reaction. While compound **19** can be obtained from **20** in 5 steps, compound **18** is easily accessible from readily available propargyl alcohol. Compound **20** in turn can be synthesized from commercially available 1,8-octanediol in a 3-step sequence (Scheme 1).

### Synthesis of strongylodiols H and I

**Scheme 2:**
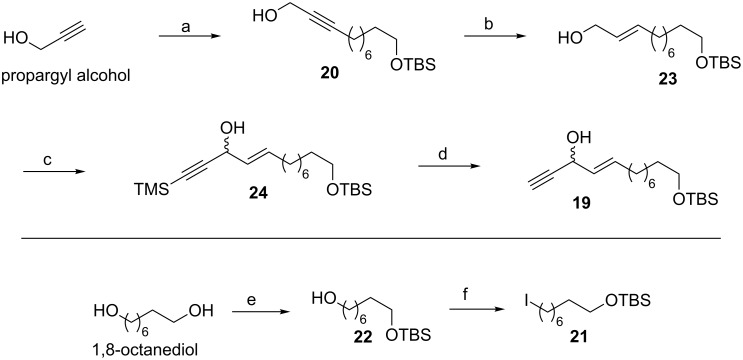
Synthesis of alkyne **19** and iodo intermediate **21**. Reagents and conditions: (a) *n*-BuLi, THF, −78 °C to rt, **21**, 12 h, 72%; (b) Red-Al, anh. Et_2_O, −20 °C, 8 h, 95%; (c) (i) DMSO, CH_2_Cl_2_, (COCl)_2_, −78 °C, (ii) TMS-C*≡*CH, *n*-BuLi, THF, −78 °C to rt, 3 h, 89%; (d) K_2_CO_3_, MeOH, 0 °C to rt, 1 h, 92%; (e) TBSCl, imidazole, 0 °C to rt, 1 h, 98%; (f) I_2_, TPP, imidazole, 0 °C to rt, 1 h, 92%.

The synthesis began with the C-alkylation reaction of propargyl alcohol with *tert*-butyl((8-iodooctyl)oxy)dimethylsilane [14] (**21**, prepared from 1,8-octanediol in two steps) in the presence of *n*-BuLi to provide alkynol **20** in 72% yield. The *trans*-selective reduction [25] of alkyne **20** was easily achieved with sodium bis(2-methoxyethoxy)aluminum hydride (Red-Al) as a hydride-transfer reagent to furnish the corresponding (*E*)*-*allylic alcohol **23**. A Swern oxidation of **23** provided the corresponding aldehyde which was subjected to an addition reaction with lithium(trimethylsilyl)acetylide to get the coupled product **24**. The latter compound on further treatment with K_2_CO_3_ in MeOH [26] furnished the desilylated propargylic alcohol **19** (Scheme 2).

**Scheme 3:**
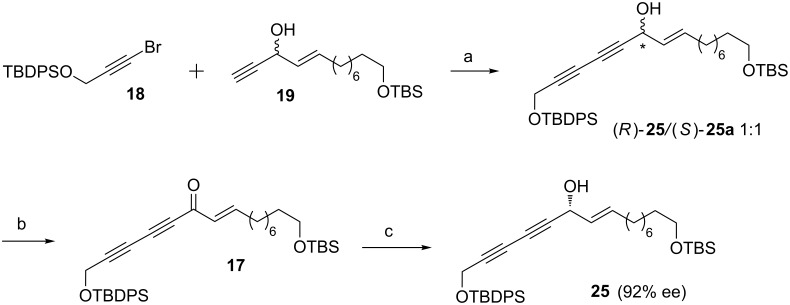
Stereoselective synthesis of (*R*)-**25**. Reagents and conditions: (a) CuCl, NH_2_OH·HCl, 30% *n*-BuNH_2_, Et_2_O, 1 h, 68%; (b) DMP, CH_2_Cl_2_, 0 °C to rt, 1 h, 87%; (c) (*S*)-CBS catalyst, BH_3_·DMS, THF, −50 °C, 16 h, 85%.

The copper(I)-catalyzed Cadiot–Chodkiewicz [27] cross-coupling reaction between bromoalkyne **18** [28] and terminal alkyne **19** provided the corresponding diynes **25** and **25a** in a 1:1 ratio. Though we had an option to proceed further with the mixture of **25** and **25a** affording both enantiomers that could be separated later, we focused our attention towards the synthesis of the required chiral compound. Thus, the mixture of **25** and **25a** was oxidized under Dess–Martin conditions to give the prochiral ene–yne–one **17** in 87% yield. The ketone **17** was then subjected to a stereoselective asymmetric reduction [23,29–31] in the presence of (*S*)-CBS as the catalyst to yield the chiral propargylic alcohol **25** with 92% ee (Scheme 3) [32].

**Figure 2:**
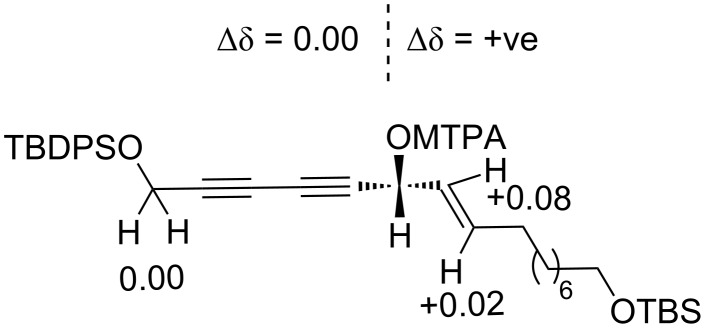
Absolute configuration analysis of alcohol **25**. ∆δ = δ_S_ − δ_R_ for the (*R*)- and (*S*)-MTPA ester of alcohol **25**.

After derivatization and determination through Mosher’s ester analysis [33–35], the absolute configuration of the newly generated secondary hydroxy group-bearing carbon center (C6) was determined as R configuration (see Supporting Information File 1). The NMR analysis of the Mosher’s esters showed a positive chemical shift difference ∆δ for the protons at C7 and C8 indicating the *R* configuration at C6 in compound **25** (see Figure 2).

**Scheme 4:**
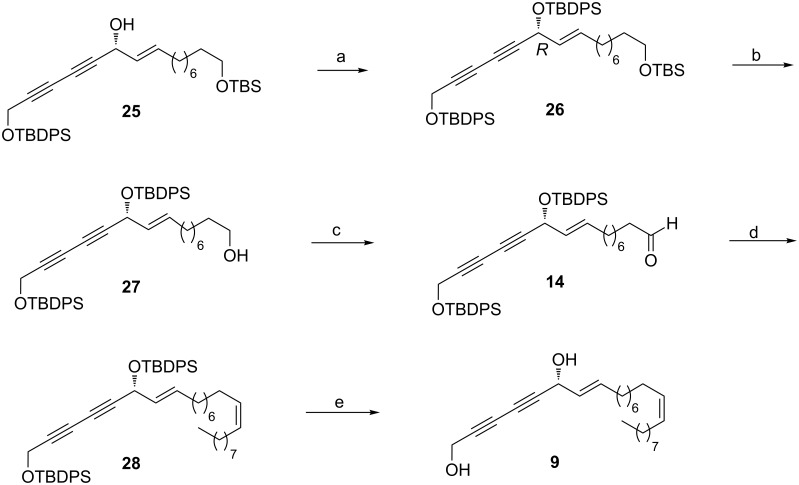
Synthesis of strongylodiol H (**9**). Reagents and conditions: (a) TBDPSCl, imidazole, CH_2_Cl_2_, 0 °C to rt, 2 h, 95%; (b) PPTS, MeOH, 0 °C to rt, 2 h, 95%; (c) IBX, THF/DMSO, 0 °C to rt, 1 h, 97%; (d) **15**, *n*-BuLi, THF, −78 °C to rt, 2 h, 83%; (e) TBAF, THF, 0 °C to rt, 2 h, 85%.

Having determined the stereochemistry at the newly generated carbon center in intermediate **25**, we proceeded further to extend the chain at the right hand side. Towards this, the free secondary hydroxy group in **25** was masked as its corresponding TBDPS ether **26** and then treated with PPTS in MeOH [36] to afford the disilylated primary alcohol **27** in 95% yield. Treatment of alcohol **27** with IBX [37] furnished the corresponding aldehyde **14** which was subjected to Wittig olefination reaction with triphenylphosphonium salt of *n-*nonyl bromide **15** in presence of *n*-BuLi to produce the corresponding *Z-*olefin **28** exclusively in 83% yield. Di-desilylation of compound **28** was easily achieved with *n*-tetrabutylammonium fluoride to furnish the target molecule strongylodiol H **9** in 85% yield (Scheme 4).

**Figure 3:**
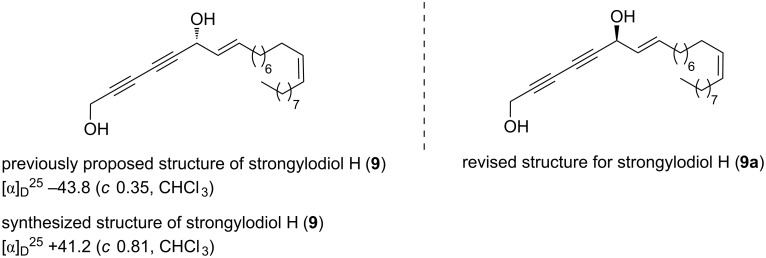
Previously proposed and revised structure of strongylodiol H (**9**).

Though the ^1^H NMR and ^13^C NMR spectra of the synthesized product were in full agreement with that of the reported natural product [10], the specific rotation of our synthetic product was determined as [α]_D_^25^ = +42.2 (*c* 0.81, CHCl_3_), and for the natural product it was found to be [α]_D_^25^ = −43.8 (*c* 0.35, CHCl_3_) [10]. Having identical spectral data (see Table 1 for comparative ^1^H and ^13^C NMR data for the synthetic and natural strongylodiol H), but with the opposite sign of rotation, and based on the outcome of our synthesis, we unambiguously revise the structure of the natural product as **9a** which is the enantiomer of the proposed structure **9** (Figure 3).

**Table 1:** Comparison of ^1^H and ^13^C NMR data of strongylodiol H (isolated natural product vs synthetic).

position	^1^H NMR for isolated compound [10] (CDCl_3_, 500 MHz)	^1^H NMR for synthesized compound (CDCl_3_, 400 MHz)	^13^C NMR for isolated compound [10] (CDCl_3_, 125 MHz)	^13^C NMR for synthesized compound (CDCl_3_, 100 MHz)

1	4.36 (d, 6.3)	4.35 s	51.5	51.42
2	–	–	77.9	77.96
3	–	–	69.8	69.78
4	–	–	69.8	69.75
5	–	–	78.7	78.66
6	4.89 (brt, 6.2)	4.89 (d, 5.8)	63.3	63.29
7	5.57 (dd, 6.2, 15.3)	5.57 (dd, 6.2, 15.3)	127.7	127.65
8	5.89 (td, 6.7, 15.3)	5.93–5.85 m	135.2	135.19
9	2.06 (q, 6.7)	2.14–1.97 m	31.9	31.96
10	1.39 m	1.49–1.21 m	28.8	28.76
11–13	1.24–1.31 m		–	–
14	1.34 m		28.7	28.76
15	2.01 (q, 6.1)	2.14–1.97 m	27.18	27.19
16	5.35 m	5.37–5.32 m	129.8	129.79
17			130.0	129.95
18	2.01 (q, 6.1)	2.14–1.97 m	27.21	27.19
19	2.14 (t, 6.7)		29.8	29.72
20–22	1.24–1.31 m	1.49–1.21 m	–	–
23	1.24–1.31 m		32.0	31.96
24	1.24–1.31 m		22.7	22.67
25	0.88 (t, 6.7)	0.88 (t, 6.7)	14.1	14.10

**Scheme 5:**
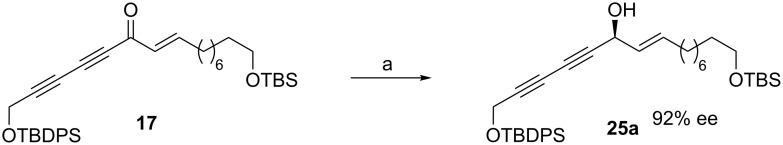
Synthesis of compound **25a**. Reagents and conditions: (a) (*R*)-CBS catalyst, BH_3_·DMS, −50 °C, 16 h, 86%.

Further, to reconfirm the structural revision, we synthesized the other enantiomer of strongylodiol H. Towards this we proceeded for the stereoselective reduction of prochiral ketone **17** with (*R*)-CBS as the catalyst furnishing the chiral propargylic alcohol **25a** with 92% ee (Scheme 5).

**Scheme 6:**
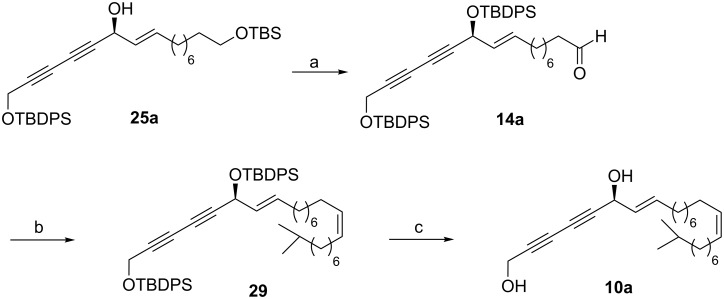
Synthesis of strongylodiol I (**10a**). Reagents and conditions: (a) (i) TBDPSCl, imidazole, CH_2_Cl_2_, 0 °C to rt, 1 h, 96%; (ii) PPTS, MeOH, 0 °C to rt, 2 h, 87%; (iii) IBX, DMSO/THF 1:1, 0 °C to rt, 1 h, 98%; (b) **16**, *n*-BuLi, THF, −78 °C to rt, 2 h, 81%; (c) TBAF, THF, 0 °C to rt, 2 h, 82%.

After securing the absolute configuration at C6 in compound **25a** by employing the Mosher^’^s ester analysis indicating *S-*configuration (see Supporting Information File 1), compound **25a** was subjected to TBDPS protection followed by TBS deprotection to give the primary alcohol which was subjected to oxidation to yield aldehyde **14a** (Scheme 6). The latter was then subjected to a Wittig reaction with **15** followed by silyl deprotection as performed for **14** to furnish **9a** (see Supporting Information File 1 for experimental details). The sign of the optical rotation for **9a** {[α]_D_^25^ = −40.2 (*c* 0.72, CHCl_3_)} was found to be in accordance with that reported for the isolated natural product {[α]_D_^25^ = −43.8 (*c* 0.35, CHCl_3_)} [10]. Thus, the total synthesis of the natural product (−)-strongylodiol H has been accomplished successfully.

As the proposed structure for strongylodiol H was revised, we next turned our attention to the total synthesis of strongylodiol I, having an additional methyl group attached to carbon atom C24. Towards this, aldehyde **14a** was subjected to a Wittig reaction with the triphenylphosphonium salt of 1-iodo-8-methylnonane **16**, prepared earlier in our group [24], to furnish the corresponding *Z*-olefin **29** exclusively in 81% yield. Exposure of **29** to *n*-tetrabutylammonium fluoride provided the natural product (*S*)-strongylodiol I (**10a**) in 82% yield (Scheme 6). The analytical data and specific rotation of the synthetic product were found to be comparable with the previously reported data of the isolated product {[α]_D_^25^ = −30.2 (*c* 1.7, CHCl_3_)}; Lit. {[α]_D_^25^ = −33.4 (*c* 0.35, CHCl_3_)} [10] (see Table 2 for comparative ^1^H and ^13^C NMR data for the synthetic and natural strongylodiol I). Thus, our synthesis has led us to revise the proposed structure of strongylodiol I as **10a**. Based on these findings we believe, that the structures of other strongylodiols E, F, G and J may have to be revised accordingly as they contain only one similar chiral center as observed in strongylodiols H and I.

**Table 2:** Comparison of ^1^H and ^13^C NMR data of strongylodiol I (isolated natural product vs synthetic).

position	^1^H NMR for isolated compound [10] (CDCl_3_, 500 MHz)	^1^H NMR for synthesized compound (CDCl_3_, 500 MHz)	^13^C NMR for isolated compound [10] (CDCl_3_, 125 MHz)	^13^C NMR for synthesized compound (CDCl_3_, 125 MHz)

1	4.35 (d, 5.8)	4.34 s	51.5	51.42
2	–	–	77.9	77.95
3	–	–	69.80	69.76
4	–	–	68.83	69.76
5	–	–	78.7	78.66
6	4.89 (brt, 6.0)	4.89 (d, 5.9)	63.3	63.29
7	5.51 (brdd, 6.0, 15.3)	5.57 (dd, 6.2, 15.3)	127.7	127.66
8	5.90 (td, 6.8, 15.3)	5.93–5.85 m	135.2	135.19
9	2.06 (q, 6.8)	2.10–1.97 m	32.0	31.96
10	1.39 m	1.57–1.11 m	28.8	28.75
11–13	1.24–1.30 m		–	–
14	1.34 m		29.74	28.73
15	2.02 (q, 6.1)	2.10–1.97 m	27.19	27.18
16	5.34 m	5.38–5.31 m	129.8	129.79
17			130.0	129.95
18	2.02 (q, 6.0)	2.10–1.97 m	27.22	27.20
19	1.34 m	1.57–1.11 m	29.77	29.79
20–21	1.24–1.30 m		–	–
22	1.24–1.31 m		27.4	27.36
23	1.15 m		39.0	39.02
24	1.52 m		28.0	27.94
25–26	0.86 (d, 6.6)	0.86 (d, 6.7)	22.7	22.64

## Conclusion

In conclusion, we have accomplished the first enantioselective total synthesis of the two diacetylenic diol natural products strongylodiol H and strongylodiol I and of an enantiomer of strongylodiol H. Our synthesis assisted us to revise the structure of both natural products strongylodiol H and I. The synthetic procedure involved the alkylation of the commercially available propargyl alcohol, a Cadiot–Chodkiewicz cross coupling, and CBS catalyzed reduction of the intermediate ene–yne–one as the key steps. The natural products strongylodiol H and I were obtained in 16.2%, 15.4% yields, respectively, from the commercially available propargyl alcohol involving 13 linear steps, respectively. Investigations towards exploring the current strategy for accessing other natural products and their analogues are currently underway.

## Supporting Information

File 1 Experimental details and analytical data.

File 2 ^1^H NMR and ^13^C NMR spectra of key compounds.
