# Supplementary material for: Stereoselective total synthesis and structural revision of the diacetylenic diol natural products strongylodiols H and I
Source: Beilstein J Org Chem. 2018 Sep 4;14:2313–20. doi: 10.3762/bjoc.14.206 (PMC6142741; doi:10.3762/bjoc.14.206)
Supplement: File 1 — Experimental details and analytical data. [file Beilstein_J_Org_Chem-14-2313-s001.pdf]

Supporting Information File 1

for

Stereoselective total synthesis and structural revision of the diacetylenic diol natural products strongylodiols H and I

Pamarthi Gangadhar[1], Sayini Ramakrishna[1], Ponneri Venkateswarlu[2] and Pabbaraja Srihari[1,*]

Address: [1]Department of Organic Synthesis and Process Chemistry, CSIR-Indian Institute of Chemical Technology, Hyderabad-500007, Telangana, India and [2]Department of Chemistry, S. V. U. College of Sciences, Tirupati-517502

Email: Srihari Pabbaraja - srihari@iict.res.in

*Corresponding author

Experimental details and analytical data

Table of contents

Analytical data of Mosher's ester **32**, **32a**, **33** and **33a** S2

Experimental procedures S4

(*R*)-MTPA ester (31a):

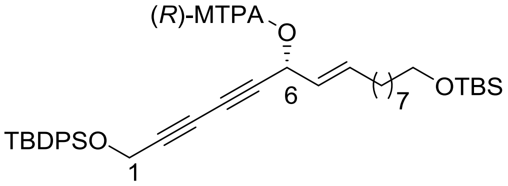

To a stirred solution of alcohol **25** (20 mg, 0.032 mmol), DCC (13.3 mg, 0.064 mmol) and DMAP (0.78 mg, 0.0006 mmol) in CH_2Cl_2 (1.0 mL) at rt was added (+)-(*R*)-α-methoxy-α-(trifluoromethyl)-phenylacetic acid (9.8 mg, 0.042 mmol) in one portion. After 10 h, the crude reaction mixture was purified directly by flash column chromatography (10% EtOAc/hexanes) to provide the (*R*)-MTPA ester (6.2 mg, 23%, 0.0074 mmol). ^{1}H NMR (500 MHz, $CDCl_3$): δ 7.72–7.66 (m, 4H), 7.53–7.37 (m, 11H), 6.12 (dd, *J* = 6.8, 16.01 Hz, 1H), 5.67 (d, *J* = 15.8 Hz, 1H), 5.47 (m, 1H), 4.40 (s, 2H), 3.60 (t, *J* = 6.6, 2H), 3.56 (s, 3H), 1.70–1.40 (m, 4H), 1.38–1.18 (m, 14H), 1.06 (s, 9H), 0.90 (s, 9H), 0.05 (s, 6H) ppm.

(*S*)-MTPA ester (31):

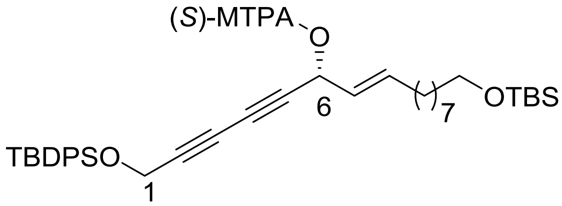

To a stirred solution of alcohol **25** (20 mg, 0.032mmol), DCC (13.3 mg, 0.064 mmol) and DMAP (0.78 mg, 0.0006 mmol) in CH_2Cl_2 (1.0 mL) at rt was added (−)-(*S*)-α-methoxy-α-(trifluoromethyl)-phenylacetic acid (9.8 mg, 0.042 mmol) in one portion. After 10 h, the crude reaction mixture was purified directly by flash column chromatography (10% EtOAc/hexanes) to provide the (*S*)-MTPA ester (7.2 mg, 27%, 0.0087 mmol). ^{1}H NMR (500 MHz, $CDCl_3$): δ 7.72–7.67 (m, 4H), 7.52–7.37 (m, 11H), 6.20 (dd, *J* = 7.0, 15.8 Hz, 1H), 5.79 (d, *J* = 16.7 Hz, 1H), 5.49 (m, 1H), 4.40 (s, 2H), 3.60 (t, *J* = 6.6, 2H), 3.54 (s, 3H), 1.64–1.44 (m, 4H), 1.34–1.19 (m, 14H), 1.06 (s, 9H), 0.90 (s, 9H), 0.05 (s, 6H) ppm.

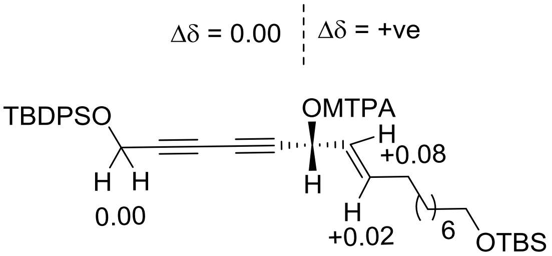

	δ (ppm) (*S*)-Mosher ester	δ (ppm) (*R*)-Mosher ester	$\Delta\delta_{SR}$ (= $\delta_S - \delta_R$) (500 MHz)
1 ^{1}H	4.40	4.40	0.00
7 ^{1}H	6.20	6.12	0.08
8 ^{1}H	5.49	5.47	0.02

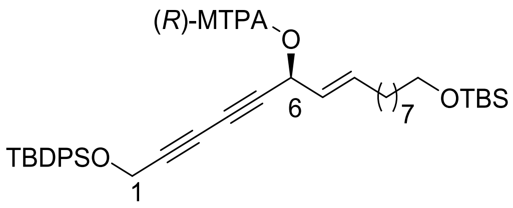

(*R*)-MTPA ester (32a): To a stirred solution of alcohol **25a** (18 mg, 0.029 mmol), DCC (12 mg, 0.058 mmol) and DMAP (0.7 mg, 0.0005 mmol) in CH_2Cl_2 (1.0 mL) at rt was added (+)-(*R*)-α-methoxy-α-(trifluoromethyl)-phenylacetic acid (8.8 mg, 0.037 mmol) in one portion. After 10 h, the crude reaction mixture was purified directly by flash column chromatography (10% EtOAc/hexanes) to provide the (*R*)-MTPA ester of **33a** (8 mg, 35%, 0.010 mmol). ^{1}H NMR (500 MHz, $CDCl_3$): δ 7.71–7.67 (m, 4H), 7.52–7.37 (m, 11H), 6.20 (dd, *J* = 7.1, 15.8 Hz, 1H), 5.79 (d, *J* = 16.0 Hz, 1H), 5.48 (m, 1H), 4.40 (s, 2H), 3.58 (t, *J* = 6.7, 2H), 3.54 (s, 3H), 1.69–1.43 (m, 1H), 1.33–1.14 (m, 16H), 1.06 (s, 9H), 0.89 (s, 9H), 0.05 (s, 6H) ppm.

(*S*)-MTPA ester (32):

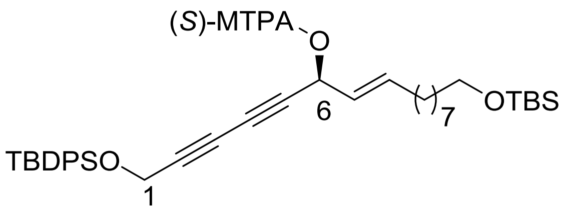

To a stirred solution of alcohol **25a** (26 mg, 0.042 mmol), DCC (17 mg, 0.084 mmol) and DMAP (1.0 mg, 0.0008 mmol) in CH_2Cl_2 (1.0 mL) at rt was added (−)-(*S*)-α-methoxy-α-(trifluoromethyl)-phenylacetic acid (12.8 mg, 0.054 mmol) in one portion. After 10 h, the crude reaction mixture was purified directly by flash column chromatography (10% EtOAc/hexanes) to provide the (*S*)-MTPA ester of **33** (7.3 mg, 21%). ^{1}H NMR (500 MHz, $CDCl_3$): δ 7.71–7.67 (m, 4H), 7.51–7.37 (m, 11H), 6.12 (dd, *J* = 6.8, 16.0 Hz, 1H), 5.67 (d, *J* = 15.8 Hz, 1H), 5.47 (m, 1H), 4.40 (s, 2H), 3.59 (t, *J* = 6.5, 2H), 3.56 (s, 3H), 1.56–1.46 (m, 1H), 1.33–1.24 (m, 15H), 1.06 (s, 9H), 0.90 (s, 9H), 0.05 (s, 6H) ppm.

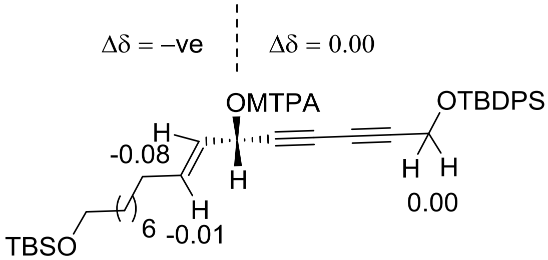

	δ (ppm) (*S*)-Mosher ester	δ (ppm) (*R*)-Mosher ester	Δ*δ*$_{SR}$ (= δ$_S$ – δ$_R$) (500 MHz)

1 ^{1}H	4.40	4.40	0.00
7 ^{1}H	6.12	6.20	-0.08
8 ^{1}H	5.47	5.48	-0.01

General experimental procedures:

^{1}H NMR and ^{13}C NMR spectra were recorded in $CDCl_3$ on 300 MHz or 500 MHz spectrometers at ambient temperature. The coupling constants *J* are given in hertz, Hz. The chemical shifts are reported in ppm on a scale downfield from TMS as internal standard and signal patterns are indicated as follows: s = singlet, d = doublet, dd = doublet of doublet, dt = doublet of triplet, t = triplet, q = quartet, qd = quartet of doublet, m = multiplet, br = broad. FTIR spectra were recorded in $CHCl_3$ and are reported in wave numbers (cm^{-1}). For low (MS) and high (HRMS) resolution mass spectra, *m/z* ratios are reported as values in atomic mass units. Mass analysis was done in the ESI mode. Optical rotations were measured on an Anton Paar digital polarimeter and the values given are specific rotations. All reagents were reagent grade and used without further purification unless specified otherwise. Solvents for reactions were distilled prior to use: THF, toluene and diethyl ether were distilled from Na and benzophenone ketyl; MeOH from Mg and I_2; CH_2Cl_2 from CaH_2. All air or moisture-sensitive reactions were conducted under a nitrogen or argon atmosphere in flame-dried or oven-dried glassware with magnetic stirring. Reactions were monitored by thin layer chromatography carried out on silica plates (silica gel 60 F254, Merck) using UV light, iodine and anisaldehyde for visualization. Column chromatography was carried out using silica gel (60–120 mesh or 100–200 mesh) packed in glass columns. Technical grade ethyl acetate and petroleum ether used for column chromatography were distilled prior to use.

8-((*tert*-Butyldimethylsilyl)oxy)octan-1-ol (22):

Imidazole (7.6 g, 112 mmol) was added to a solution of 1,8-octanediol (15.0 g, 102 mmol) in CH_2Cl_2 (200 mL) at 0 °C. The solution was stirred for 15 min before adding TBSCl (23.1g, 154 mmol) at 0 °C and allowed to warm to rt. After 1 h, the reaction mixture was diluted with cold water (200 mL), the aqueous layer extracted with CH_2Cl_2 (2 × 150 mL) and dried over anhydrous Na_2SO_4. Removal of the solvent under reduced pressure afforded a colorless oil that was purified by silica gel (60–120 mesh) column chromatography to afford **22** as colorless oil (26.1 g, 100.6 mmol, 98%); R_f = 0.5 (20%, EtOAc-hexane).

IR ν max: 3345, 2929, 2856, 1466, 1387, 1252, 1096, 773, 662 $cm^{-1.}$

^{1}H NMR (400 MHz, $CDCl_3$): δ 3.56 (dt, *J*=2.8, 6.7 Hz, 4H), 1.55–1.41 (m, 1H), 1.34–1.21 (m, 12H), 0.85 (s, 9H), 0.01 (s, 6H) ppm.

^{13}C NMR (100 MHz, CDCl$_3$): δ 63.22, 62.67, 32.73, 32.62, 29.34, 29.31, 25.88, 25.64, 18.26, -5.36 ppm.

MS(ESI): *m/z* 261 $[M+H]^+$.

HRMS(ESI) *m/z* calculated for $C_{14}H_{32}O_2Si$ 261.2244, found 261.2242.

***tert*-Butyl((8-iodooctyl)oxy)dimethylsilane (21):** To the solution of compound **22** (15 g, 57.6 mmol) in THF, imidazole (11.7 g, 172 mmol), triphenylphosphine (30.23 g, 115 mmol) and iodine (32.1 g, 126 mmol) were added at 0 °C and the reaction mixture was stirred for 1 h until the alcohol was completely consumed as determined by TLC. The reaction mixture was quenched with saturated aq. hypo solution and extracted with Et_2O (3 × 100 mL). The organic layer was dried over magnesium sulfate and concentrated under reduced pressure to afford a dark oil. The crude product was purified by column chromatography (60–120 silica gel) using a gradient of 20 to 40% EtOAc in hexane to give **21** (19.6 g, 53.0 mmol, 92%) as a colorless oil.

IR ν max: 2927, 2855, 1462, 1388, 1360, 1252, 1214, 1195, 772, 661 cm^{-1}.

^{1}H NMR (300 MHz, CDCl$_3$): δ 3.60 (t, *J* = 6.7 Hz, 2H), 3.18 (t, *J* = 7.09 Hz, 2H), 1.86–1.78 (m, 2H), 1.53–1.47 (m, 1H), 1.36–1.26 (m, 8H), 0.89 (s, 9H), 0.05 (s, 6H) ppm.

^{13}C NMR(100 MHz, CDCl$_3$): δ 62.22, 33.52, 32.78, 30.43, 30.31, 29.19, 28.51, 25.97, -5.25 ppm.

MS(ESI): *m/z* 371 $[M+H]^+$.

11-((*tert*-Butyldimethylsilyl)oxy)undec-2-yn-1-ol (20): A solution of *n*-BuLi (1.6 M in hexane, 33.4 mL, 53.5 mmol) was added to a THF solution (10 mL) of propargylic alcohol (1.5 g, 26.7 mmol) at −78 °C under argon atmosphere, and the mixture was stirred for 30 min. To this solution was added the solution of compound **21** (15.9 g, 53.5 mmol) and the mixture was warmed to rt and stirred for 12 h. The reaction was quenched by adding aq. saturated NH_4Cl (50 mL) at 0 °C, and the aqueous phase was extracted with diethyl ether (4 × 60 mL). The organic layer was washed with brine (100 mL), and dried over $MgSO_4$. After removal of the solvent, compound **20** (5.7 g, 72%, 19.2 mmol) was purified through 60–120 mesh silica gel column chromatography. R_f = 0.5 (20% EtOAc-Hexane).

IR υ max: 3368, 2930, 2857, 1465, 1387, 1361, 1252, 1219, 833, 773 cm^{-1}.

^{1}H NMR (500 MHz, CDCl$_3$): δ 4.24 (s, 2H), 3.59 (t, *J* = 6.6 Hz, 2H), 2.23–2.18 (m, 2H), 1.54–1.56 (m, 5H), 1.45–1.26 (m, 8H), 0.89 (s, 9H), 0.04 (s, 6H) ppm.

^{13}C NMR (125 MHz, $CDCl_3$): δ 86.52, 78.31, 77.19, 63.25, 51.36, 32.74, 28.85, 28.76, 28.48, 25.96, 25.63, 18.68, -5.28 ppm.

MS(ESI): *m/z* 321 $[M+Na]^+$.

HRMS(ESI) *m/z* calculated for $C_{17}H_{34}O_2Si$ 299.2401, found 299.2410.

(*E*)-11-((*tert*-Butyldimethylsilyl)oxy)undec-2-en-1-ol (23): Compound **20** (4.0 g, 13.4 mmol) was dissolved in anhydrous diethyl ether (250 mL) and RED-Al (10.8 mL, 33.5 mmol, 65% solution in toluene) was added at −20 °C. The mixture was stirred at room temperature under nitrogen for 8 h. Then, the reaction was quenched by careful dropwise addition of water, then diluted further with water (250 mL) and extracted with ethyl acetate (3 × 100 mL). The combined organic extract was dried over $MgSO_4$. Filtration followed by solvent evaporation afforded the crude product **23** which was purified by silica gel column chromatography to give (3.8 g, 95%, 12.7 mmol). R_f = 0.5 (20% EtOAc-Hexane).

IR ν max: 3359, 2927, 2856, 1465, 1362, 1252, 1095, 1004, 969, 774, 661 cm^1.

^{1}H NMR (500 MHz, $CDCl_3$): δ 5.74–5.09 (m, 2H), 4.08 (d, *J* = 5.5 Hz, 2H), 3.59 (d, *J* = 6.6 Hz, 2H), 2.04 (q, *J* = 6.4 Hz, 2H),1.53–1.46 (m, 2H), 1.40–1.26 (m, 11H), 0.89 (s, 9H), 0.04 (s, 6H) ppm.

^{13}C NMR (125 MHz, $CDCl_3$): δ 133.52, 128.82, 63.84, 63.28, 32.83, 32.16, 29.24, 29.11, 29.04, 25.97, 25.71, 18.36, -5.26 ppm.

HRMS(ESI) *m/z* calculated for $C_{17}H_{36}O_2$ NaSi 323.2382, found 323.2370.

(*R*),(*S*)-(*E*)-13-((*tert*-Butyldimethylsilyl)oxy)-1-(trimethylsilyl)tridec-4-en-1-yn-3-ol (24): A solution of oxalyl chloride (2.0 mL, 23.2 mmol) in dry CH_2Cl_2 (20 mL) was cooled to −78 °C under an atmosphere of argon. A solution of DMSO (3.3 mL, 46.4 mmol) in CH_2Cl_2 (20 mL) was added at a rate such that the reaction temperature remained below −65 °C. After stirring for 5 min, a solution of **23** (3.5 g, 11.6 mmol) in CH_2Cl_2 (35 mL) was added slowly, and the resulting mixture was stirred for 15 min. Triethyl amine (9.7 mL, 69.6 mmol) was added slowly and after stirring the mixture for additional 10 min at −78 °C, the cooling bath was removed and the reaction was allowed to warm to room temperature over 45 min with stirring. Water (50 mL) was added and stirring was continued for additional 15 min. Then, the reaction mixture was washed successively with saturated $NaHCO_3$ solution (50 mL), and brine (50 mL). The organic layer was dried (Na_2SO_4), filtered, and concentrated under reduced pressure to afford an oily aldehyde, which was directly used in the next step without further purification.

A solution of *n*-BuLi in hexane (1.6 M, 16.7 mL, 26.8 mmol) was added dropwise to a stirred and cooled solution of TMS acetylene (3.75 mL, 26.8 mmol) in dry THF (30 mL) at −78 °C under an argon atmosphere. To the resulting solution was added crude aldehyde (4.0 g, 13.4 mmol in 30 mL THF) in a dropwise manner. The reaction mixture was stirred for 1 h at same temperature, and then allowed to warm to room temperature over the next 2 h. The mixture was then diluted with ice flakes and aq. NH_4Cl solution, and extracted with ethyl acetate (4 × 50 mL). The organic layer was washed with water and brine, dried ($MgSO_4$), concentrated in vacuum and purified by silica gel (60–120 mesh) column chromatography to give **24** (inseparable enantiomeric mixture) as a yellow liquid (4.7 g, 11.9 mmol, 89%). R_f = 0.3 (20% EtOAc-Hexane).

IR ν max: 3367, 2929, 2857, 1465, 1385, 1251, 1097, 1027, 965, 764, 661 cm^1.

^{1}H NMR (400MHz, CDCl$_3$): δ 5.91–5.84 (m, 1H), 5.61–5.55 (m, 1H), 4.81 (d, *J* = 6.1 Hz, 1H), 3.59 (t, *J* = 6.5 Hz, 2H), 2.09–2.03 (m, 2H), 1.53–1.47 (m, 3H), 1.42–1.36 (m, 5H), 1.34–1.27 (m, 5H), 0.89 (s, 9H), 0.18 (s, 6H), 0.04 (s, 6H) ppm.

^{13}C NMR (100 MHz, CDCl$_3$): δ 134.25, 128.63, 104.91, 90.56, 63.39, 63.27, 32.83, 31.90, 29.23, 28.77, 25.97, 25.72, 18.37, 0.15, -5.25 ppm.

MS(ESI): *m/z* 419 $[M+Na]^+$.

HRMS(ESI) *m/z* calculated for $C_{22}H_{44}O_2Si_2Na$ 419.2772, found 419.2773.

(*R*),(*S*)-(*E*)-13-((*tert*-Butyldimethylsilyl)oxy)tridec-4-en-1-yn-3-ol (19): To a suspension of K_2CO_3 (3.45 g, 24.9 mmol) in MeOH (25 mL) cooled at 0 °C and maintained under a N_2 atmosphere was added a solution of **24** (4.5 g, 11.3 mmol) in MeOH (30 mL) dropwise. The reaction mixture was stirred at room temperature for 1 h, during which time a large amount of a white turbid precipitate had formed. The reaction mixture was diluted with EtOAc (50 mL) and quenched with crushed ice pieces. The organic layer was washed with H_2O, brine, dried over anhydrous Na_2SO_4 and filtered. The solvent was removed under vacuum and the residue was purified by flash chromatography on silica gel using 10% EtOAc/hexane as the eluent to afford compound **19** as colorless oil (3.38 g, 10.4 mmol) in 92% yield. R_f = 0.4(20% EtOAc-Hexane).

IR ν max: 3368, 2930, 2857, 1465, 1384, 1251, 1217, 1096, 965, 836, 663 cm^1.

^{1}H NMR (300 MHz, CDCl$_3$): δ 5.95–5.88 (m, 1H), 5.63–5.58 (m, 1H), 4.83 (d, *J* = 5.6 Hz, 1H), 3.59 (t, *J* = 6.5 Hz, 2H), 2.56 (d, *J* = 2.2 Hz, 1H), 2.06 (q, *J* = 7.0 Hz, 2H), 1.53–1.47 (m, 2H), 1.42–1.36 (m, 2H), 1.34–1.28 (m, 9H), 0.89 (s, 9H), 0.04 (s, 6H) ppm.

^{13}C NMR (75 MHz, CDCl$_3$): δ 134.50, 128.37, 83.33, 73.92, 62.78, 32.81, 31.88, 29.21, 29.11, 28.74, 25.97, 25.69, 18.37, -5.27 ppm.

HRMS(ESI) *m/z* calculated for $C_{19}H_{37}O_2Si$ 325.2563, found 325.2561.

***tert*-Butyldiphenyl(prop-2-yn-1-yloxy)silane (30):** To a stirred solution of imidazole (7.2 g, 107.1 mmol) and propargylic alcohol (2.0 g, 35.7 mmol) in CH_2Cl_2 (30 mL) at 0 °C was added *t*-$BuPh_2SiCl$ (10.08 mL, 39.2 mmol) and the reaction mixture was allowed to warm to rt. After 1 h, the reaction mixture was diluted with water (50 mL) and the aq. layer was extracted with CH_2Cl_2 (3 × 50 mL), and dried over $MgSO_4$. Removal of the solvent under reduced pressure afforded a crude oil that was purified through silica gel (60–120 mesh) column chromatography to afford *tert*-butyldiphenyl(prop-2-yn-1-yloxy)silane (**30**) as a colorless oil (9.6 g, 32.8 mmol 92%); R_f = 0.4 (10 % EtOAc-Hexane).

IR ν max: 3298, 3071, 2957, 2932, 2893, 2859, 1589, 1468, 1369, 739cm^{-1}**.**

^{1}H NMR (500 MHz, CDCl$_3$) δ 7.79–7.75 (m, 4H), 7.50–7.41 (m, 6H), 4.37 (d, *J* = 2.4 Hz, 2H), 2.42 (t, *J* = 2.4 Hz, 1H), 1.13 (s, 9H) ppm.

^{13}C NMR (125 MHz) (CDCl$_3$): δ 135.55, 132.92, 129.80, 127.71, 81.97, 73.03, 52.44, 26.26 ppm.

HRMS(ESI) *m/z* calculated for $C_{19}H_{23}OSi$ 295.1518, found 295.1508.

((3-Bromoprop-2-yn-1-yl)oxy)(*tert*-butyl)diphenylsilane(18): To the solution of *tert*-butyldiphenyl(prop-2-yn-1-yloxy)silane (**30**, 4.0 g, 13.6 mmol) dissolved in acetone (30 mL) was added NBS (4.84 g, 27.2 mmol) and silver nitrate (4.62 g, 27.2 mmol). The reaction mixture was stirred at rt for 1 h. Then the reaction mixture was cooled to 0 °C, diluted with cold water, and extracted with Et_2O (3 × 40 mL). The combined organic layer was dried over anhydrous Na_2SO_4 and concentrated under reduced pressure. The residue was purified by silica gel chromatography (60–120 mesh, 1% EtOAc/hexane) to afford compound **18** (4.15 g, 82%, 11.1 mmol) as yellow oil.

IR ν max: 3070, 2957, 2932, 2893, 2858, 2219, 1736, 1589, 1468, 1427, 821, 615 cm^{-1}.

^{1}H NMR (300 MHz, CDCl$_3$): δ 7.75–7.71 (m, 4H), 7.49–7.40 (m, 6H), 4.35 (s, 2H), 1.09 (s, 9H) ppm.

^{13}C NMR (75 MHz, CDCl$_3$): δ 135.56, 132.80, 129.83, 127.71, 78.20(2C), 53.41, 26.65 ppm.

HRMS(ESI) *m/z* calculated for $C_{19}H_{22}OSiBr$ 373.0623, found 373.0637.

(*R*),(*S*)-(*E*)-2,2,22,22,23,23-Hexamethyl-3,3-diphenyl-4,21-dioxa-3,22-disilatetracosa-11-en-6,8-diyn-10-ol (25 + 25a): CuCl (10 mg) was added to a 30% *n*-butylamine solution (15 mL) to get a blue-colored solution. After the addition of a few crystals of hydroxylamine hydrochloride, the blue color disappeared. Then the alkyne **19** (3.0 g, 9.25 mmol) in 30 mL diethyl ether was added at once and the mixture was immediately cooled to 0 °C. Then, the bromo alkyne **18** (3.09 g, 8.33 mmol) was added at once and the mixture stirred for 60 min. During the reaction, additional hydroxylamine hydrochloride crystals (to maintain lower oxidation state of copper) had to be added in appropriate intervals. Afterwards, the reaction mixture was extracted with diethyl ether (5 × 40 mL), the combined organic layer dried over anhydrous Na_2SO_4 and concentrated under reduced pressure. The crude was purified through silica gel (60–120 mesh) column chromatography to afford a 1:1 enantiomeric mixture of **25** + **25a** (3.87 g, 68%, 6.2 mmol). R_f = 0.4 (20% EtOAc-Hexane).

(*E*)-2,2,22,22,23,23-Hexamethyl-3,3-diphenyl-4,21-dioxa-3,22-disilatetracosa-11-en-6,8-diyn-10-one (17): Analogous to the description in [23], to Dess–Martin periodinane (5.16 g, 12.1 mmol) placed in a round-bottomed flask was added a solution of alcohols **25** + **25a** (3.0 g, 4.8 mmol) in dichloromethane (40 mL) at 0 °C. The reaction was allowed to stir at rt for 30 min. After the reaction was complete as determined by TLC, the formed solid was removed by filtration from the reaction mixture through a small pad of celite and washed with dichloromethane twice. The filtrate was washed sequentially with aqueous sodium bicarbonate and brine and dried over anhydrous $MgSO_4$. The solvent was removed under reduced pressure to afford a solid residue that was purified through silica gel (60–120 mesh) column chromatography to afford the corresponding propargylic ketone **17** (2.6 g, 4.2 mmol, 87%) as colorless liquid. R_f = 0.3 (10% EtOAc-Hexane).

IR ν max: 3071, 2930, 2856, 2234, 2156, 1643, 1466, 1286, 1149, 660 cm^{-1}.

^{1}HNMR (500MHz, $CDCl_3$): δ 7.71–7.66 (m, 4H), 7.47–7.38 (m, 6H), 7.20–7.12 (m, 1H), 6.18 (dt, *J* = 1.4, 15.7 Hz, 1H), 4.43 (s, 2H), 3.60 (t, *J* = 6.4 Hz, 2H), 2.35–2.26 (m, 2H), 1.57–1.47 (m, 5H), 1.38–1.29 (m, 7H), 1.07 (s, 9H), 0.89 (s, 9H), 0.05 (s, 6H) ppm.

^{13}C NMR (125 MHz, $CDCl_3$): δ 177.20, 155.72, 135.54, 132.38, 132.10, 130.03, 127.84, 85.05, 75.03, 73.70, 68.21, 63.19, 53.02, 32.71, 29.19, 29.14, 27.78, 26.61, 25.97, 25.66, 1918, 18.36, -5.29 ppm.

MS(ESI): *m/z* 615 $[M+H]^+$.

(*R*,*E*)-2,2,22,22,23,23-Hexamethyl-3,3-diphenyl-4,21-dioxa-3,22-disilatetracosa-11-en-6,8-diyn-10-ol (25): Analogous to the description in [23], to a magnetically stirred solution of (*S*)-Me-CBS (1 M solution

in toluene, 1.95 mL, 1.95 mmol) in anhydrous THF (5 mL) was added dropwise BH_3·DMS (2 M solution in THF, 1.95 mL, 3.9 mmol) at 0 °C, and the reaction mixture was stirred at rt for 30 min. The generated complex-containing solution was cooled to −50 °C, and then a solution of ketone **17** (0.6 g, 0.97 mmol) in THF (6 mL) was added dropwise. The stirring was continued until the complete consumption of starting material was observed as indicated by TLC; approximately 16 h). Then the reaction mixture was quenched with a saturated aqueous solution of NH_4Cl and diluted with water (5 mL). The resulting solution was extracted with EtOAc (4 × 5 mL). The combined extracts were washed with brine (7 mL), dried with anhydrous Na_2SO_4, filtered, and concentrated to give the crude product which was purified by column chromatography to afford propargylic alcohol **25** (0.511 g, 0.83 mmol, 85%) as colorless liquid. R_f = 0.4 (20% EtOAc-Hexane).

$[\alpha]^{25}_D$= +23.31 (*c* 2.6, $CHCl_3$).

IR ν max: 3369, 3071, 2929, 2856, 1466, 1428, 1367, 1253, 1189, 738, 664 cm^{-1}.

^{1}H NMR (500 MHz, $CDCl_3$): δ 7.72–7.67 (m, 4H), 7.47–7.37 (m, 6H), 5.94–5.85 (m, 1H), 5.62–5.55 (m, 1H), 4.89 (d, *J* = 5.3 Hz, 1H), 4.37 (s, 2H), 3.60 (t, *J* = 6.6 Hz, 2H), 2.07 (q, *J* = 6.9 Hz, 2H), 1.55–1.47 (m, 2H), 1.43–1.26 (m, 11H), 1.06 (s, 9H), 0.90 (s, 9H), 0.05 (s, 6H) ppm.

^{13}C NMR (125 MHz, $CDCl_3$): δ 135.54, 135.01, 132.66, 129.89, 127.77, 78.31, 77.98, 70.19, 68.95, 63.33, 63.27, 53.00, 32.82, 31.95, 29.20, 28.71, 29.14, 26.62, 25.98, 25.69, 19.16, 18.37, -5.25 ppm.

MS(ESI): *m/z* 639 $[M+Na]^+$.

HRMS(ESI) *m/z* calculated for $C_{38}H_{56}NaO_3Si_2$ 639.3660, found 639.3661.

(*R,E*)-10-((*tert*-Butyldiphenylsilyl)oxy)-2,2,22,22,23,23-hexamethyl-3,3-diphenyl-4,21-dioxa-3,22-disilatetracosa-11-en-6,8-diyne (26): Imidazole (0.033 g, 0.486 mmol) was added to a solution of alcohol **25** (0.1 g, 0.162 mmol) in CH_2Cl_2 (5 mL) at 0 °C and stirred for 15 min before adding *t*-$BuPh_2SiCl$ (0.04 mL, 0.178 mmol) at the same temperature. The reaction mixture was allowed to warm to rt. After 1 h, the reaction mixture was diluted with water (5 mL) and the aqueous layer was extracted with CH_2Cl_2 (3 × 5 mL) and dried over ($MgSO_4$). Removal of the solvent under reduced pressure afforded a crude oil which was purified through silica gel (60–120 mesh) column chromatography affording compound **26** as pale yellow oil (0.131 g, 0.154 mmol 95%); R_f = 0.2 (10 % EtOAc-Hexane).

$[\alpha]^{25}_D$= -49.96 (*c* 0.24, $CHCl_3$).

IR ν max: 2929, 2856, 1727, 1471, 1427, 1390, 1361, 1254, 1187, 1006, 771cm^{-1}.

^{1}HNMR (500 MHz, $CDCl_3$): δ 7.77–7.65 (m, 8H), 7.46–7.34 (m, 12H), 5.60–5.45 (m, 2H), 4.80 (d, *J* = 5.8 Hz, 1H), 4.36 (s, 2H), 3.60 (t, *J* = 6.6 Hz, 2H), 2.01–1.94 (m, 2H), 1.55–1.48 (m, 1H), 1.34–1.23 (m, 11H), 1.09 (s, 9H), 1.07 (s, 9H), 0.90 (s, 9H), 0.06 (s, 6H) ppm.

^{13}C NMR (125 MHz, $CDCl_3$): δ 135.94, 135.84, 135.55, 133.44, 133.31, 133.06, 132.71, 129.86, 129.76, 129.73, 128.37, 127.75, 127.52, 78.87, 77.69, 77.19, 69.51, 69.34, 64.92, 63.27, 60.37, 53.05, 32.86, 31.87, 29.27, 29.13, 28.76, 26.82, 26.63, 25.98, 25.73, 19.29, 19.17, 18.37, -5.25 ppm.

HRMS(ESI) *m/z* calculated for $C_{54}H_{74}NaO_3Si_3$ 877.4843, found 877.4905.

(*R*,*E*)-11,16-bis((*tert*-Butyldiphenylsilyl)oxy)hexadeca-9-en-12,14-diyn-1-ol (27): Compound **26** (0.130 mmol, 0.15 mmol) was dissolved in absolute methanol (3 mL) and to this was added PPTS (45 mg, 0.18 mmol) in one portion. The reaction mixture was stirred at rt for 2 h, after which the solvent was removed and the residue dissolved in ethyl acetate. The organic solution was washed with saturated aqueous brine, water and then dried over anhydrous Mg_2SO_4. The solvent was evaporated in vacuum and the crude product was purified through silica gel (60–120 mesh) column chromatography to afford compound **27** as a colorless liquid (0.1 g, 0.135 mmol 95%); R_f = 0.4 (20%, EtOAc-Hexane).

$[\alpha]^{25}_{D}$= -53.92 (*c* 0.7, $CHCl_3$).

IR ν max: 3368, 3071, 2929, 2856, 1734, 1589, 1467, 1427, 1367, 1002, 938, 821 cm^{-1}.

^{1}H NMR (400 MHz, $CDCl_3$): δ 7.77–7.66 (m, 8H), 7.47–7.34 (m, 12H), 5.61–5.47 (m, 2H), 4.81 (d, *J* = 5.7 Hz, 1H), 4.37 (s, 2H), 3.65 (t, *J* = 6.7 Hz, 2H), 2.02–1.95 (m, 2H), 1.62–1.53 (m, 2H), 1.41–1.22 (m, 10H), 1.10 (s, 9H), 1.08 (s, 9H) ppm.

^{13}C NMR (100 MHz, $CDCl_3$): δ 135.92, 135.82, 135.54, 133.33, 133.02, 132.68, 129.86, 129.76, 129.72, 128.44, 127.74, 127.52, 127.51, 78.85, 77.70, 69.52, 69.33, 64.90, 63.01, 53.04, 32.72, 31.80, 29.19, 29.01, 28.67, 26.80, 26.62, 25.63, 19.29, 19.15 ppm.

MS(ESI): *m/z* 763 $[M+Na]^+$.

HRMS(ESI) *m/z* calculated for $C_{48}H_{60}NaO_3Si_2$ 763.3971, found 763.3969.

(*R*,*E*)-11,16-bis((*tert*-Butyldiphenylsilyl)oxy)hexadeca-9-en-12,14-diynal (14): To the alcohol **27** (0.1 g, 0.135 mmol) dissolved in a solvent mixture of THF/DMSO 1:1 (4 mL), was added at once IBX (0.094 g, 0.337 mmol) 0 °C and stirred for 1 h at rt. The reaction mixture was diluted with ice cold water (5 mL) and extracted with CH_2Cl_2 (5 × 15 mL). The combined organic layer was washed with saturated $NaHCO_3$ (10 mL), dried over anhydrous Na_2SO_4 and concentrated under reduced pressure to yield the aldehyde (0.096 g, 0.131 mmol) as light green oil in 97% yield. R_f = 0.2 (20%, EtOAc-Hexane).

^{1}HNMR (400 MHz, $CDCl_3$): δ 9.76 (t, 1H, *J* = 1.8 Hz) 7.78–7.66 (m, 8H), 7.48–7.35 (m, 12H), 5.61–5.47 (m, 2H), 4.82 (d, *J* = 5.7 Hz, 1H), 4.38 (s, 2H), 2.42 (dt, *J* = 1.8, 7.3 Hz, 2H), 2.02–1.96 (m, 2H), 1.62–1.53 (m, 2H), 1.38–1.23 (m, 8H), 1.09 (s, 9H), 1.08 (s, 9H) ppm.

(*R*)-2,2,13,13-Tetramethyl-5-((1*E*,10*Z*)-nonadeca-1,10-dien-1-yl)-3,3,12,12-tetraphenyl-4,11-dioxa-3,12-disilatetradeca-6,8-diyne (28): (*n*-Nonyl)triphenylphosphonium bromide (**15**, 0.25 g, 0.54 mmol) was dissolved in dry THF (5 mL) and cooled to −78 °C. To this solution *n*-BuLi (0.25 mL, 0.40 mmol, 1.6 M) was added dropwise and the mixture stirred for 1 h at the same temperature. During this time, the reaction color changed from colorless to brick red. Then, the crude aldehyde dissolved in anhydrous THF (2 mL) was added dropwise and stirred for 2 h after allowing the reaction mixture to warm to rt. The reaction was quenched with saturated ammonium chloride (2 mL) at 0 °C. The reaction mixture was extracted with ethyl acetate (5 × 4 mL), dried over anhydrous Na_2SO_4 and concentrated under reduced pressure. The resulting crude was purified through silica gel (60–120 mesh) column chromatography to afford compound **28** as pale yellow liquid (0.094 g, 0.115 mmol, 83%). R_f = 0.2 (10% EtOAc-Hexane).

$[\alpha]^{25}_D$= -39.90 (*c*,1.05, $CHCl_3$).

IR ν max: 3071, 3048, 3000, 2925, 2854, 1665, 1589, 1462, 1370, 1188, 998, 822, 610 cm^{-1}.

^{1}H NMR (500 MHz, $CDCl_3$): δ 7.76–7.65 (m, 8H), 7.47–7.33 (m, 12H), 5.60–5.45 (m, 2H), 5.37–5.33 (m, 2H), 4.80 (d, *J* = 5.8 Hz, 1H), 4.36 (s, 2H), 2.07–1.94 (m, 6H), 1.37–1.23 (m, 23H), 1.09 (s, 9H), 1.07 (s, 9H), 0.88 (t, *J* = 6.6, 3H) ppm.

^{13}C NMR (125 MHz, $CDCl_3$): δ 135.94, 135.84, 135.56, 133.47, 133.33, 133.08, 132.73, 129.92, 129.87, 129.81, 129.76, 129.73, 128.38, 127.76, 127.53, 78.89, 77.70, 69.52, 69.35, 64.94, 53.06, 31.89, 29.76, 29.51, 29.37, 29.31, 29.23, 29.12, 28.81, 27.20, 26.82, 26.64, 22.67, 19.30, 19.18, 14.11 ppm.

MS(ESI): *m/z* 866 $[M+NH_4]^+$.

HRMS(ESI) *m/z* calculated for $C_{57}H_{76}NaO_2Si_2$ 871.5282, found 871.5275.

(*R*,7*E*,16*Z*)-Pentacosa-7,16-dien-2,4-diyne-1,6-diol (9), (*R*)-strongylodiol H: To a solution of compound **29** (0.90 g, 0.106 mmol) in dry THF (2.0 mL) cooled to 0 °C was added *n*-tetrabutylammonium fluoride (0.44 mL, 0.44 mmol, 1.0 M) dropwise and the mixture stirred for 2 h. The reaction was quenched with saturated aqueous ammonium chloride solution (5 mL) at 0 °C. The mixture was extracted with ethyl acetate (5 × 3 mL), the combined organic extract dried over anhydrous Na_2SO_4 and concentrated under reduced pressure. The crude was purified through silica gel (60–120 mesh) column chromatography, to afford compound **9** as colorless liquid (0.033 g, 0.0902 mmol, 85%).R_f = 0.5 (20% EtOAc-Hexane). $[\alpha]^{25}_D$= +42.2 (*c* 0.81, $CHCl_3$).

IR ν max: 3308, 3003, 2921, 2853, 1717, 1663, 1457, 1336, 1306, 1085, 917, 807, 719cm^{-1}.

^{1}H NMR (400 MHz, $CDCl_3$): δ 5.93–5.85 (m, 1H), 5.57 (dd, *J* = 6.2, 15.3 Hz, 1H), 5.37–5.32 (m, 2H), 4.89 (d, *J* = 5.8 Hz, 1H), 4.35 (s, 2H), 2.14–1.97 (m, 6H), 1.49–1.21 (m, 23H), 0.88 (t, *J* = 6.7, 3H) ppm.

^{13}C NMR (100 MHz, $CDCl_3$): δ 135.19, 129.95, 129.79, 127.65, 78.66, 77.96, 69.78, 69.75, 63.29, 51.42, 31.96, 31.89, 29.72, 29.50, 29.30, 29.19, 28.76, 27.19, 27.17, 22.67, 14.10 ppm.

MS(ESI): *m/z* 395 $[M+Na]^+$.

HRMS(ESI) *m/z* calculated for $C_{25}H_{41}NaO_2$ 373.3107, found 373.3090.

(*S*,*E*)-2,2,22,22,23,23-Hexamethyl-3,3-diphenyl-4,21-dioxa-3,22-disilatetracosa-11-en-6,8-diyn-10-ol (25a): Analogous to the description in [23], to a magnetically stirred solution of (*R*)-Me-CBS (1 M solution in toluene, 1.62 mL, 1.62 mmol) in anhydrous THF (5 mL) was added dropwise BH_3·DMS (1 M solution in THF, 1.62 mL, 0.0032 mmol) at 0 °C, and the reaction mixture was stirred at rt for 30 min. The generated complex-containing solution was cooled to −50 °C and then a solution of ketone **17** (0.5 g, 0.814 mmol) in THF (5 mL) was added dropwise. The stirring was continued until the complete consumption of starting material was observed as indicated by TLC (≈ 16 h). Then, the reaction was quenched with a saturated aqueous solution of NH_4Cl and then diluted further with water (5 mL). The resulting solution was extracted with EtOAc (4 × 5 mL), the combined extracts washed with brine (7 mL), dried over anhydrous Na_2SO_4, filtered, and concentrated to give the crude product. Purification by column chromatography afforded propargylic alcohol **25a** (0.431 g, 0.70 mmol, 86%) as a colorless liquid. R_f = 0.4 (20% EtOAc-Hexane).

$[\alpha]^{25}_{D}$= -25.51 (*c*,0.49, $CHCl_3$).

(*S*,*E*)-11,16-bis((*tert*-Butyldiphenylsilyl)oxy)hexadeca-9-en-12,14-diynal (14a): Imidazole (0.033 g, 0.486 mmol) was added to a solution of alcohol **25a** (0.1 g, 0.162 mmol) in CH_2Cl_2 (5 mL) at 0 °C. After stirring the solution for 15 min *t*-$BuPh_2SiCl$ (0.04 mL, 0.178 mmol) was added at the same temperature. The reaction mixture was allowed to warm to rt and after 1 h, the reaction mixture was diluted with water (5 mL) and the aq. layer was extracted with CH_2Cl_2 (3 × 5 mL) and the combined organic layer was dried over $MgSO_4$. Removal of the solvent under reduced pressure afforded a yellow oil which was purified through silica gel (60–120 mesh) column chromatography to afford the tris-silyl ether as pale yellow oil (0.132 g, 0.155 mmol 96%); R_f = 0.2 (10 % EtOAc-Hexane).

$[\alpha]^{25}_{D}$= +45.03 (*c* 1.1, $CHCl_3$).

To the solution of the above tris-silyl ether (0.1 mmol, 0.11 mmol) in absolute methanol (3 mL) was added PPTS (35.2 mg, 0.14 mmol) in one portion at 0 °C. The reaction mixture was stirred at rt for 2 h. The solvent was removed and the residue was dissolved in ethyl acetate. The organic solution was washed with saturated aqueous brine solution, water and then dried over anhydrous Mg_2SO_4. The solvent was evaporated in vacuum and the crude product was purified through silica gel (60–120 mesh) column chromatography to afford the primary alcohol as a colorless liquid (0.075 g, 0.10 mmol 87%); R_f = 0.4 (20%, EtOAc-Hexane).

$[\alpha]^{25}_{D}$= +57.62 (*c* 0.42, $CHCl_3$).

To the above primary alcohol (0.1 g, 0.135 mmol) dissolved in the solvent mixture of THF/DMSO 1:1 (4 mL), was added at once IBX (0.094 g, 0.337 mmol) at 0 °C and the mixture stirred for 1 h at rt. Then, the reaction mixture was diluted with ice cold water (5 mL) and extracted with CH_2Cl_2 (5 × 15 mL). The combined organic layer was washed with saturated $NaHCO_3$ (10 mL), dried over anhydrous Na_2SO_4 and concentrated under reduced pressure to yield the aldehyde **14a** (0.097 g, 0.131 mmol,) as light green oil in 98% yield. R_f = 0.2 (20%, EtOAc-Hexane).

(*S*)-2,2,13,13-Tetramethyl-5-((1*E*,10*Z*)-nonadeca-1,10-dien-1-yl)-3,3,12,12-tetraphenyl-4,11-dioxa-3,12-disilatetradeca-6,8-diyne (28a): (*n*-Nonyl)triphenylphosphonium bromide (**15**, 0.177 g, 0.37 mmol) was dissolved in dry THF (4 mL) and cooled to −78 °C. To this solution *n*-BuLi (0.17 mL, 0.28 mmol, 1.6 M) was added dropwise and stirred for 1 h at the same temperature. During this time the color of the reaction mixture changed from colorless to brick red. Then, aldehyde **14a** dissolved in anhydrous THF (2 mL) was added dropwise and the mixture stirred for 2 h after allowing it to warm to rt. Then, the reaction was quenched with saturated aq. ammonium chloride (2 mL) at 0 °C and the mixture was extracted with ethyl acetate (5 × 4 mL). The combined organic layer was dried over anhydrous Na_2SO_4 and concentrated under reduced pressure. The resulting crude was purified through silica gel (60–120 mesh) column chromatography to afford compound **28a** as pale yellow liquid (0.068 g, 0.080 mmol, 85%). R_f = 0.2 (10% EtOAc-Hexane).

$[\alpha]^{25}_{D}$= +35.96 (*c* 1.1, $CHCl_3$).

(*S*,7*E*,16*Z*)-Pentacosa-7,16-dien-2,4-diyne-1,6-diol (9a), (*S*)-strongylodiol H: Compound **28a** (0.60 g, 0.07 mmol) was dissolved in dry THF (2.0 mL) and cooled to 0 °C. To this *n*-tetrabutylammonium fluoride (0.29 mL, 0.29 mmol, 1.0 M) was added dropwise and stirred for 2 h. The reaction was quenched with saturated aqueous ammonium chloride solution (3 mL) at 0 °C. The mixture was extracted with ethyl acetate (5 × 3 mL), the combined organic layer dried over anhydrous Na_2SO_4 and concentrated under

reduced pressure. The crude was purified through silica gel (60–120 mesh) column chromatography, to afford compound **9a** as colorless liquid (0.021 g, 0.058 mmol, 82%). R_f = 0.5 (20% EtOAc-Hexane).

$[\alpha]^{25}_{D}$= -40.24 (*c* 0.72, $CHCl_3$).

(*S*)-2,2,13,13-Tetramethyl-5-((1*E*,10*Z*)-18-methylnonadeca-1,10-dien-1-yl)-3,3,12,12-tetraphenyl-4,11-dioxa-3,12-disilatetradeca-6,8-diyne (29): (Isodecyl)triphenylphosphonium iodide (**16**, 0.200 g, 0.379 mmol) was dissolved in dry THF (4 mL) and cooled to −78 °C. To this *n*-BuLi (0.17 mL, 0.28 mmol, 1.6 M) was added dropwise and stirred for 1 h at the same temperature. During this the color of the reaction mixture changed from colorless to brick red. Then, the aldehyde **14a** dissolved in anhydrous THF (2 mL) was added dropwise and the resulting mixture stirred for 2 h after allowing the reaction mixture to warm to rt. The reaction was quenched with saturated aq. ammonium chloride (2 mL) at 0 °C and extracted with ethyl acetate (5 × 4 mL). The combined organic layer was dried over anhydrous Na_2SO_4 and concentrated under reduced pressure. The resulting crude was purified through silica gel (60–120 mesh) column chromatography to afford compound **29** as pale yellow liquid (0.066 g, 0.076 mmol, 81%). R_f = 0.2 (10% EtOAc-Hexane).

$[\alpha]^{25}_{D}$= +37.01 (*c* 0.77, $CHCl_3$).

IR ν max: 2925, 2856, 1463, 1431, 1368, 1104, 1078, 965, 816, 738, 700 cm^{1}.

^{1}H NMR (500 MHz, $CDCl_3$): δ 7.76–7.64 (m, 8H), 7.46–7.33 (m, 12H), 5.60–5.44 (m, 2H), 5.38–5.32 (m, 2H), 4.79 (d, *J* = 5.8 Hz, 1H), 4.36 (s, 2H), 2.06–1.93 (m, 6H), 1.60–1.47 (m, 4H), 1.36–1.21 (m, 16H), 1.18–1.13 (m, 1H), 1.08 (s, 9H), 1.06 (s, 9H), 0.86 (d, *J* = 6.6, 6H) ppm.

^{13}C NMR (125 MHz, $CDCl_3$): δ 135.94, 135.84, 135.56, 133.46, 133.07, 132.73, 129.91, 129.82, 129.85, 129.75, 129.72, 128.37, 127.75, 127.53, 78.89, 77.70, 69.52, 69.35, 64.94, 53.06, 39.03, 31.87, 29.80, 29.76, 29.37, 29.34, 29.24, 29.12, 28.81, 27.95, 27.36, 27.20, 26.82, 26.64, 22.65, 19.30, 19.18 ppm. **MS(ESI):** *m/z* $[M\text{-}H]^-$. 885.

HRMS(ESI) *m/z* calculated for $C_{58}H_{78}NaO_2Si_2Na$ 885.5438, found 885.5496.

(*S*,7*E*,16*Z*)-24-Methylpentacosa-7,16-dien-2,4-diyne-1,6-diol, strongylodiol I, (10a), (*S*)-strongylo-diol I: Compound **29** (0.60 g, 0.069 mmol) was dissolved in dry THF (2.0 mL) and cooled to 0 °C. To this *n*-tetrabutylammonium fluoride (0.29 mL, 0.29 mmol, 1.0 M) was added dropwise and stirred for 2 h. The reaction was quenched with saturated aqueous ammonium chloride solution (3 mL) at 0 °C and extracted with ethyl acetate (5 × 3 mL). The combined organic layer was dried over anhydrous Na_2SO_4 and concentrated under reduced pressure. The crude was purified through silica gel (60–120 mesh)

column chromatography, to afford compound **10a** as colorless liquid (0.021 g, 0.056 mmol, 82%).R_f = 0.5 (20% EtOAc-Hexane).

$[\alpha]^{25}_D$ = -30.21 (*c* 1.7, $CHCl_3$).

IR ν max: 3331, 3004, 2922, 2854, 1713, 1458, 1370, 1262, 1085, 807, 720 cm^{-1}.

^{1}H NMR (500 MHz, $CDCl_3$): δ 5.93–5.85 (m, 1H), 5.57 (dd, *J* = 6.2, 15.3 Hz, 1H), 5.38–5.31 (m, 2H), 4.89 (d, *J* = 5.9 Hz, 1H), 4.34 (s, 2H), 2.10–1.97 (m, 6H), 1.57–1.11 (m, 22H), 0.86 (d, *J* = 6.7, 6H) ppm.

^{13}C NMR (125 MHz, $CDCl_3$): δ 135.19, 129.95, 129.79, 127.66, 78.66, 77.95, 69.76 (2C), 63.29, 51.42, 39.02, 31.96, 29.79, 29.73, 29.33, 29.19, 29.14, 28.75, 27.94, 27.36, 27.20, 27.18, 22.64 ppm.

MS(ESI): *m/z* 409 $[M+Na]^+$.

HRMS(ESI) *m/z* calculated for $C_{26}H_{43}O_2$ 387.3263, found 387.3251.
