# Supplementary material for: Stereoselective total synthesis and structural revision of the diacetylenic diol natural products strongylodiols H and I
Source: Beilstein J Org Chem. 2018 Sep 4;14:2313–20. doi: 10.3762/bjoc.14.206 (PMC6142741; doi:10.3762/bjoc.14.206)
Supplement: File 2 — 1H NMR and 13C NMR spectra of key compounds. [file Beilstein_J_Org_Chem-14-2313-s002.pdf]

Supporting Information File 2

for

Stereoselective total synthesis and structural revision of the diacetylenic diol natural products strongylodiols H and I

Pamarthi Gangadhar[1], Sayini Ramakrishna[1], Ponneri Venkateswarlu[2] and Pabbaraja Srihari[1,*]

Address: [1]Department of Organic Synthesis and Process Chemistry, CSIR-Indian Institute of Chemical Technology, Hyderabad-500007, Telangana, India and [2]Department of Chemistry, S. V. U. College of Sciences, Tirupati-517502

Email: Srihari Pabbaraja - srihari@iict.res.in

*Corresponding author

^{1}H NMR and ^{13}C NMR spectra of key compounds

Table of contents

Copies of ^{1}H and ^{13}C NMR S2

LCMS Chromatogram of compound **33a** S21

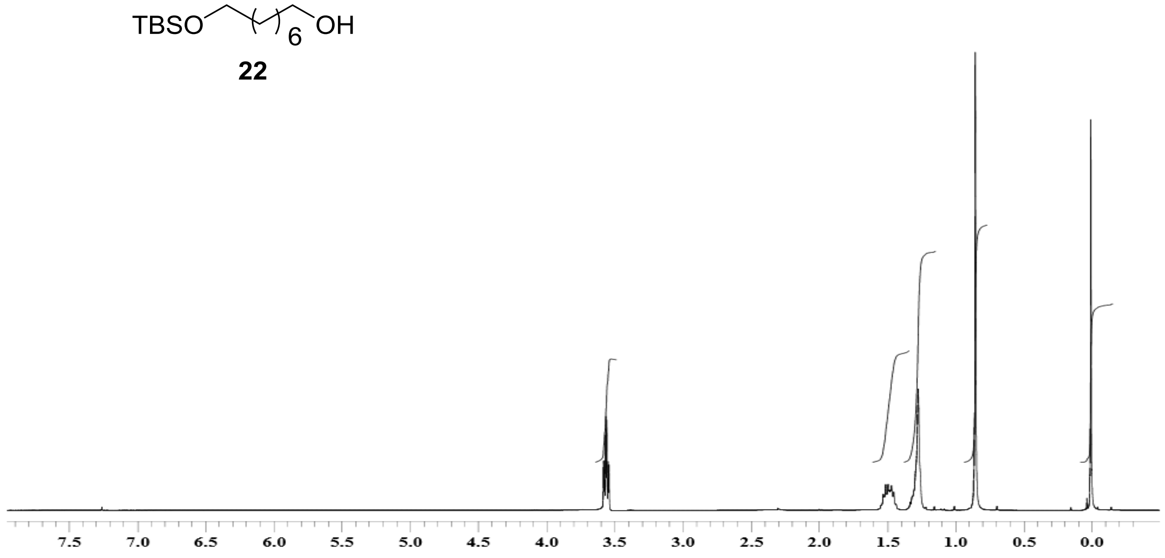

^{1}H NMR Spectrum of compound **22** ($CDCl_3$, 400 MHz)

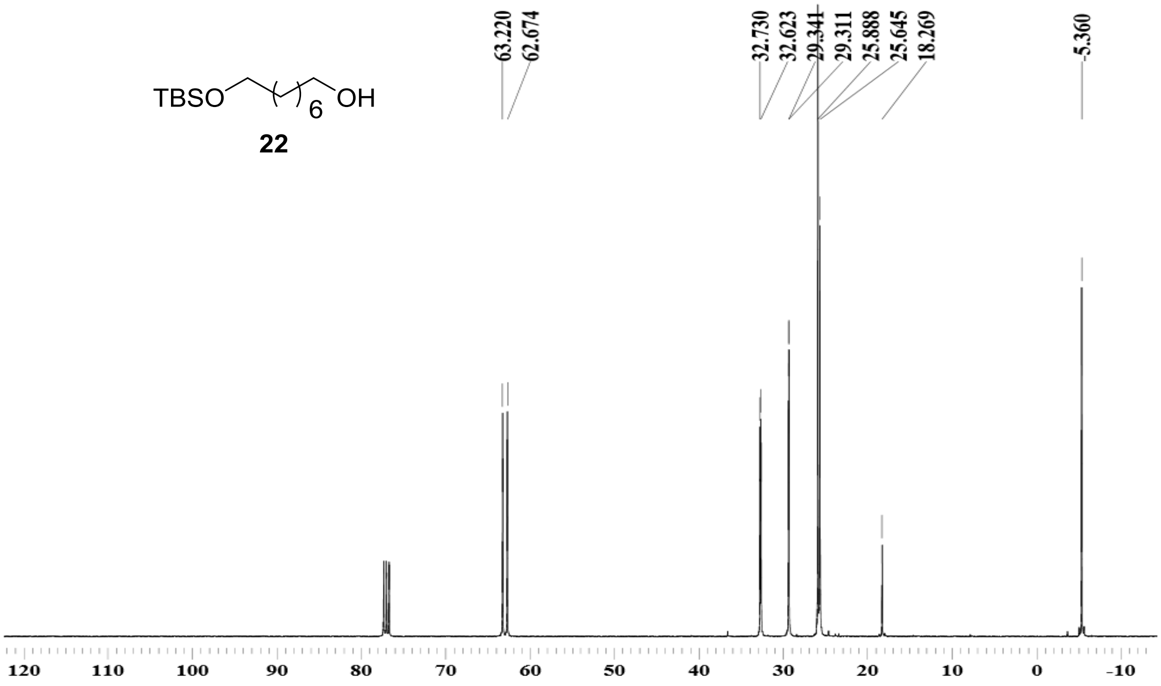

^{13}C NMR Spectrum of compound **22** ($CDCl_3$, 100 MHz)

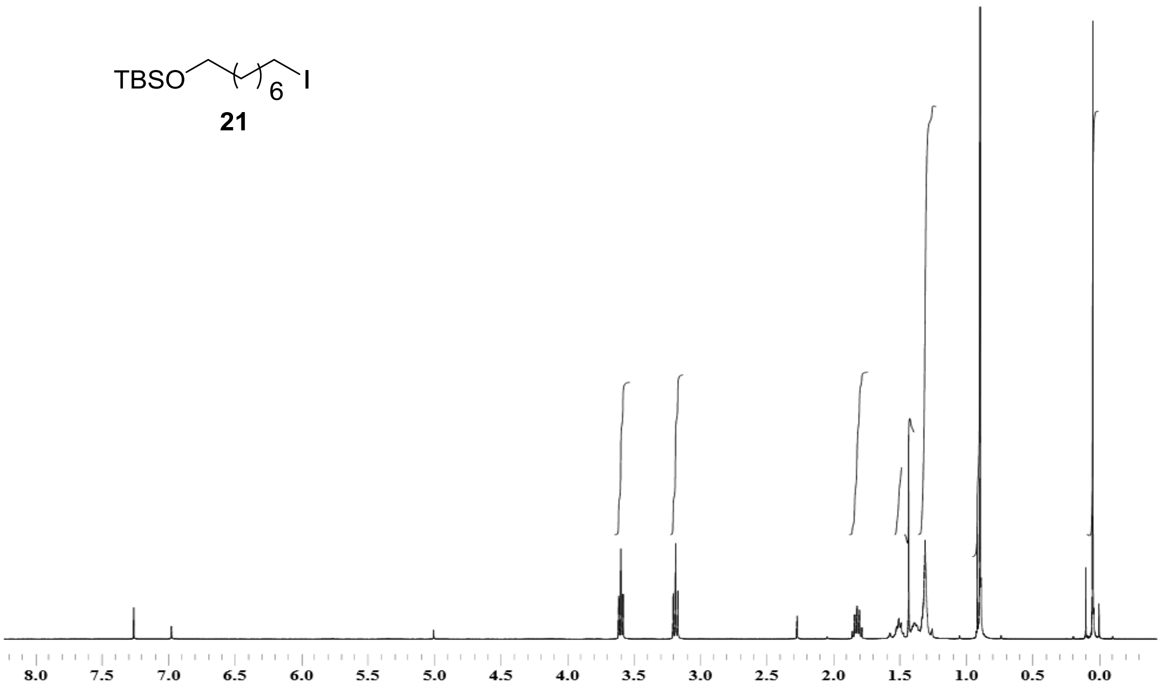

^{1}H NMR Spectrum of compound **21** ($CDCl_3$, 300 MHz)

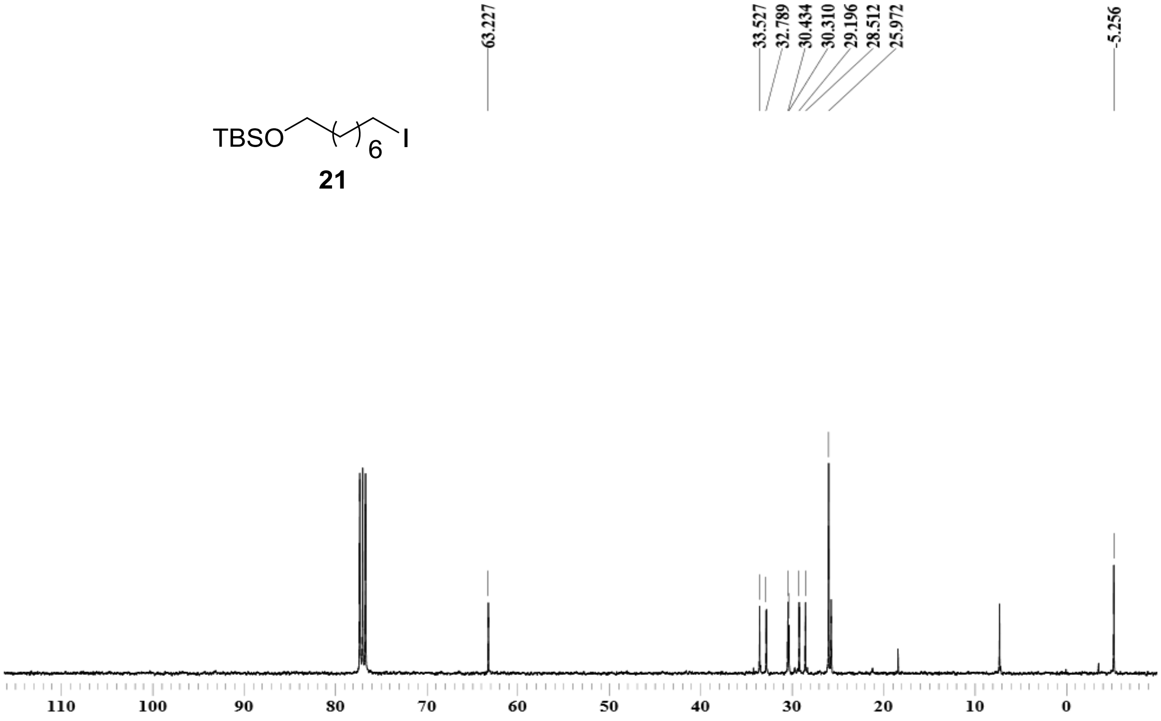

^{13}C NMR Spectrum of compound **21** ($CDCl_3$, 75 MHz)

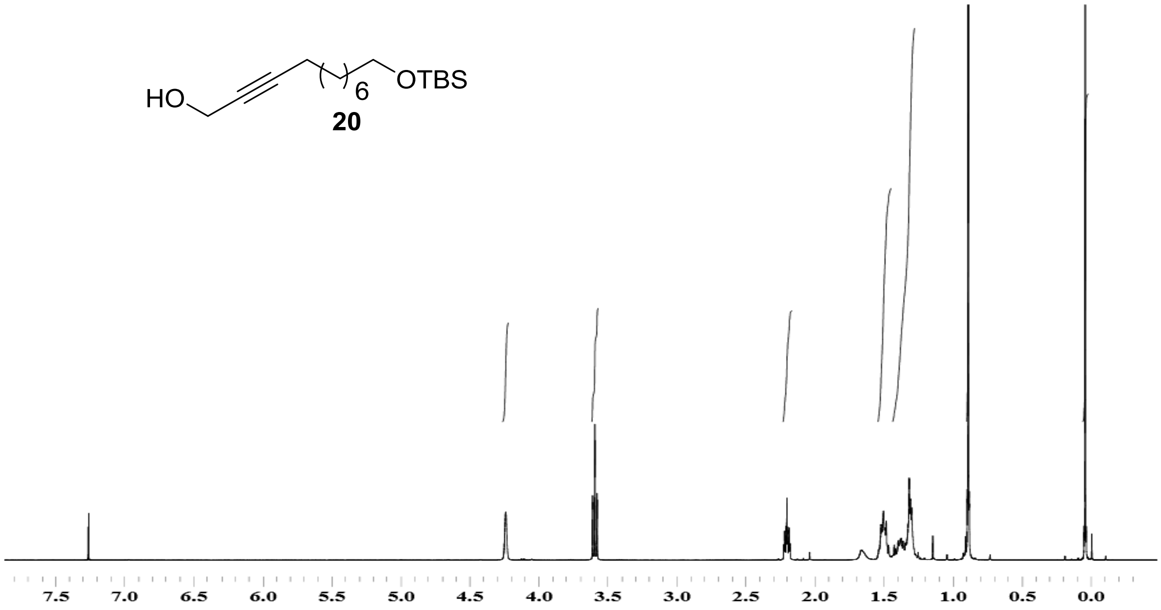

^{1}H NMR Spectrum of compound **20** ($CDCl_3$, 500 MHz)

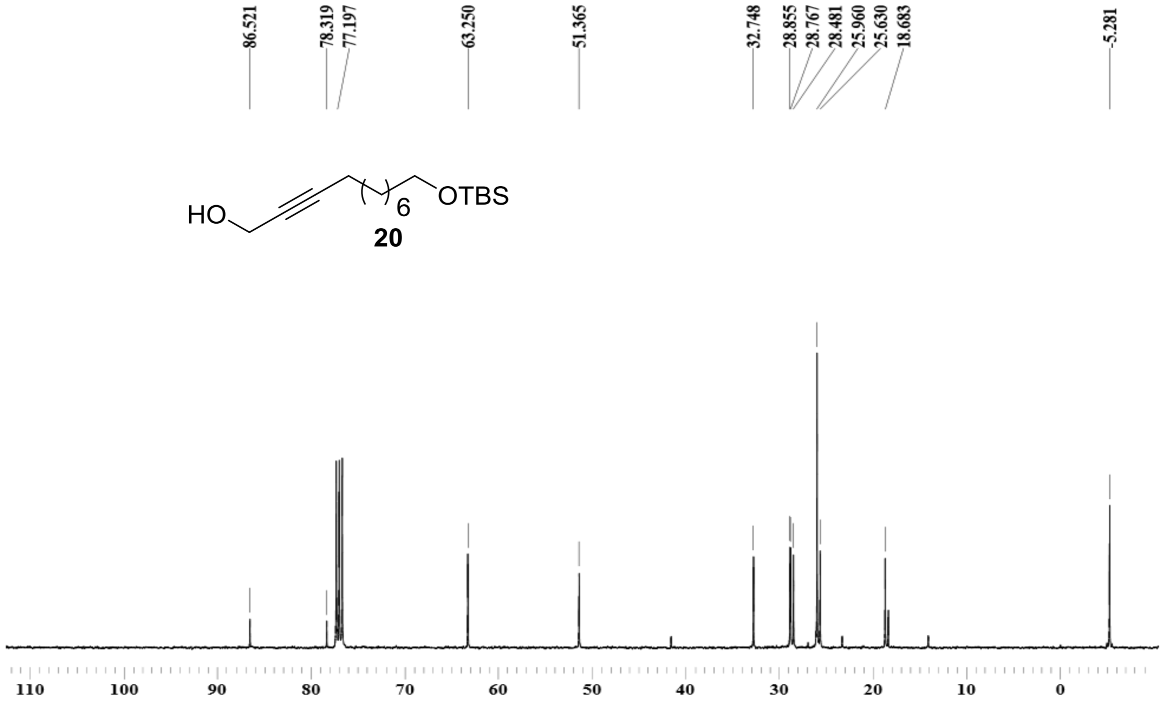

^{13}C NMR Spectrum of compound **20** ($CDCl_3$, 125 MHz)

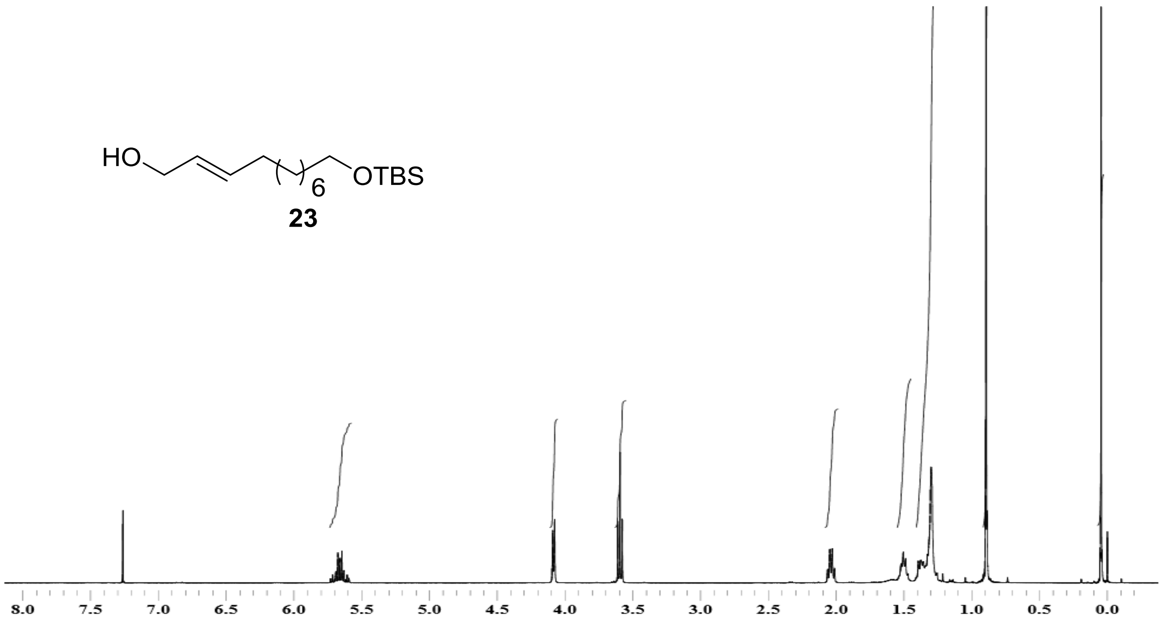

^{1}H NMR Spectrum of compound **23** ($CDCl_3$, 500 MHz)

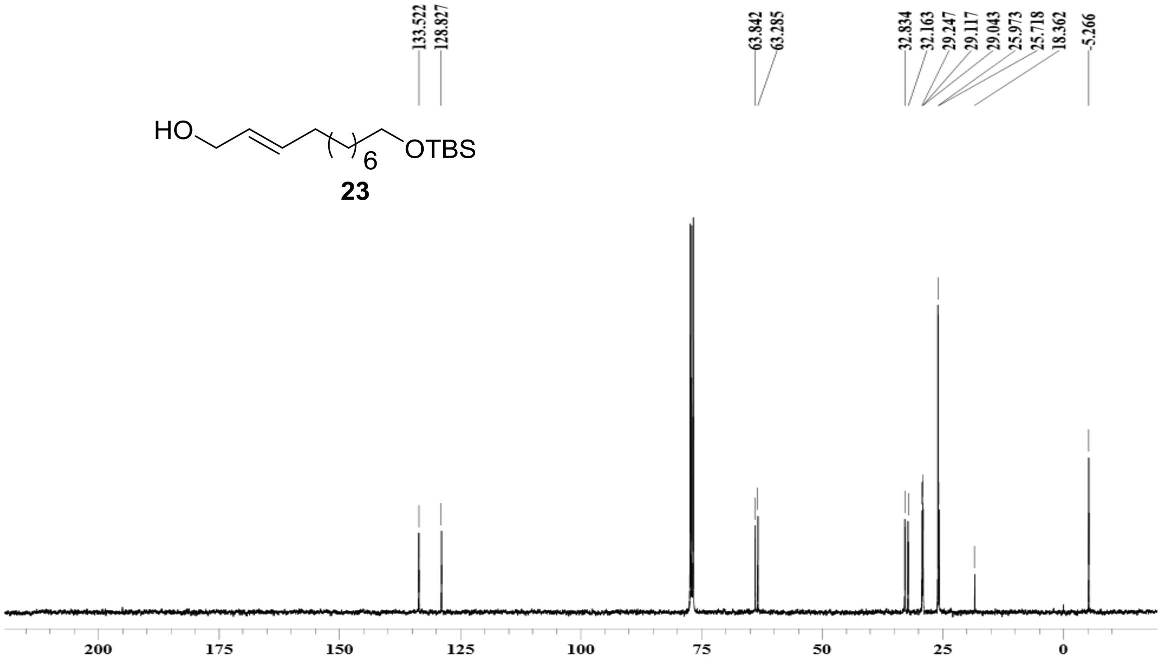

^{13}C NMR Spectrum of compound **23** ($CDCl_3$, 125 MHz)

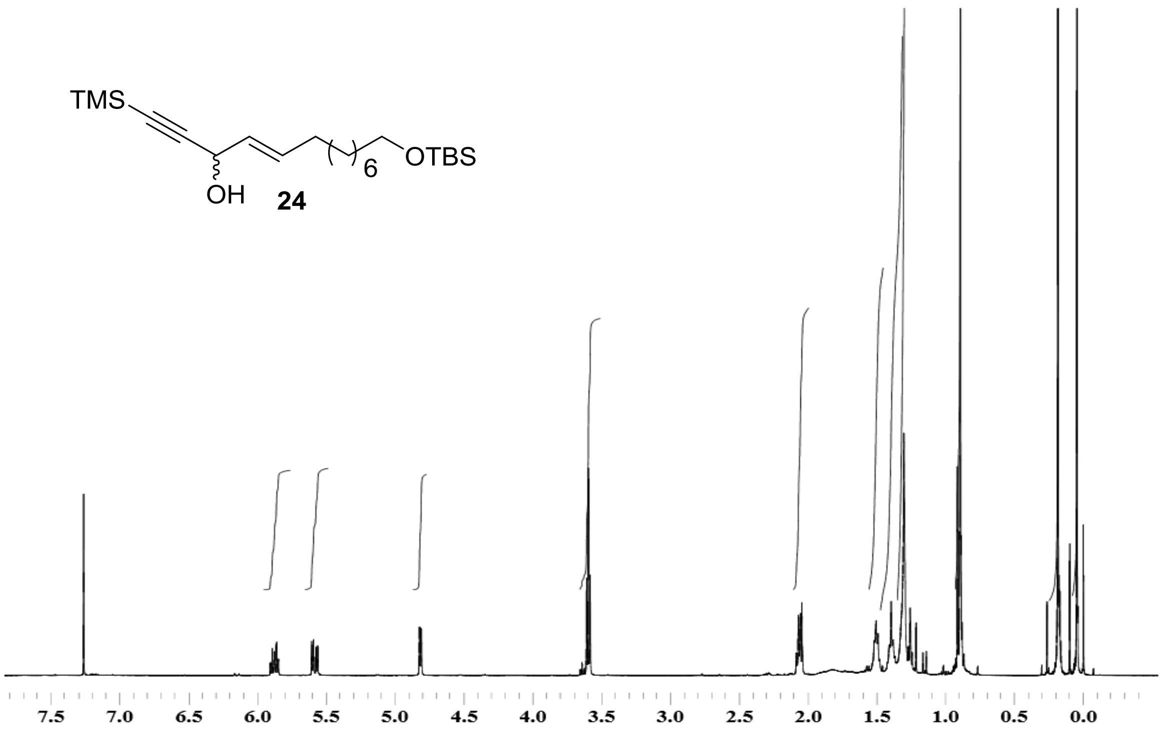

^{1}H NMR Spectrum of compound **24** ($CDCl_3$, 400 MHz)

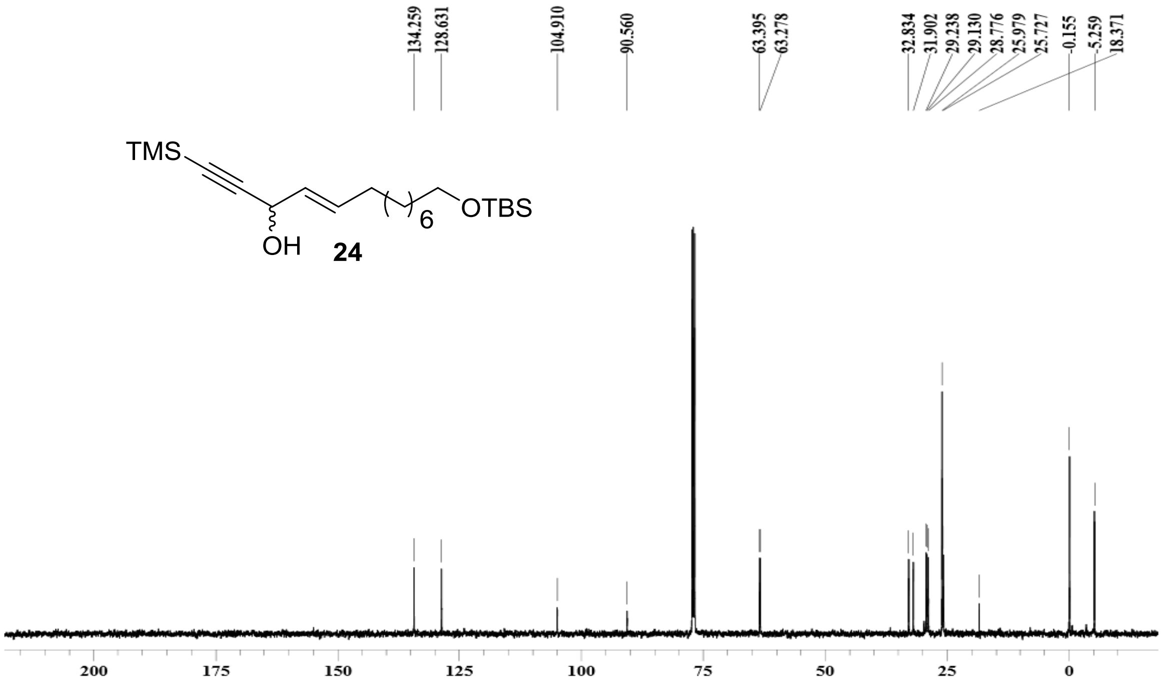

^{13}C NMR Spectrum of compound **24** ($CDCl_3$, 100 MHz)

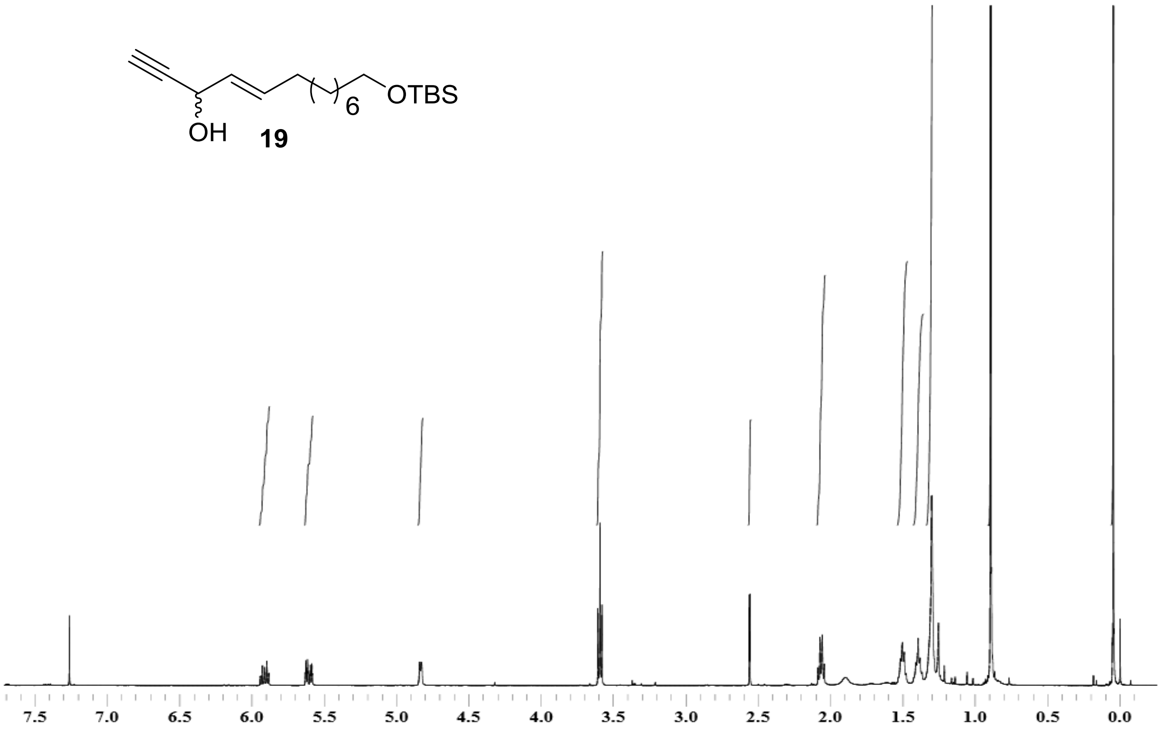

^{1}H NMR Spectrum of compound **19** ($CDCl_3$, 300 MHz)

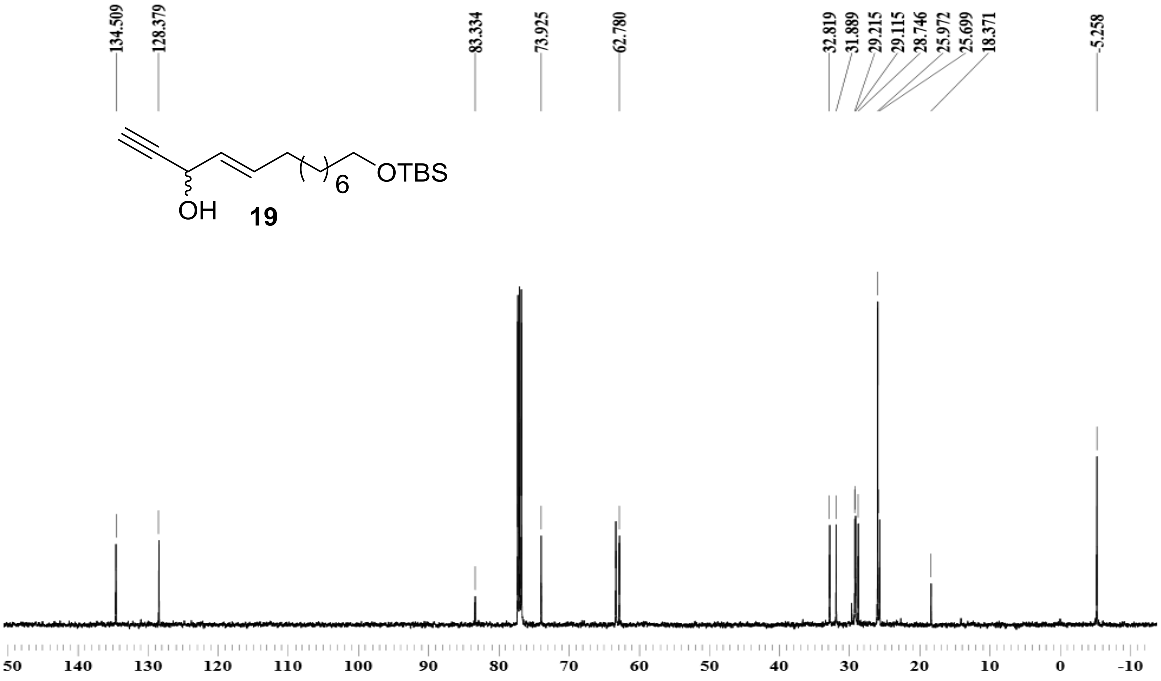

^{13}C NMR Spectrum of compound **19** ($CDCl_3$, 75 MHz)

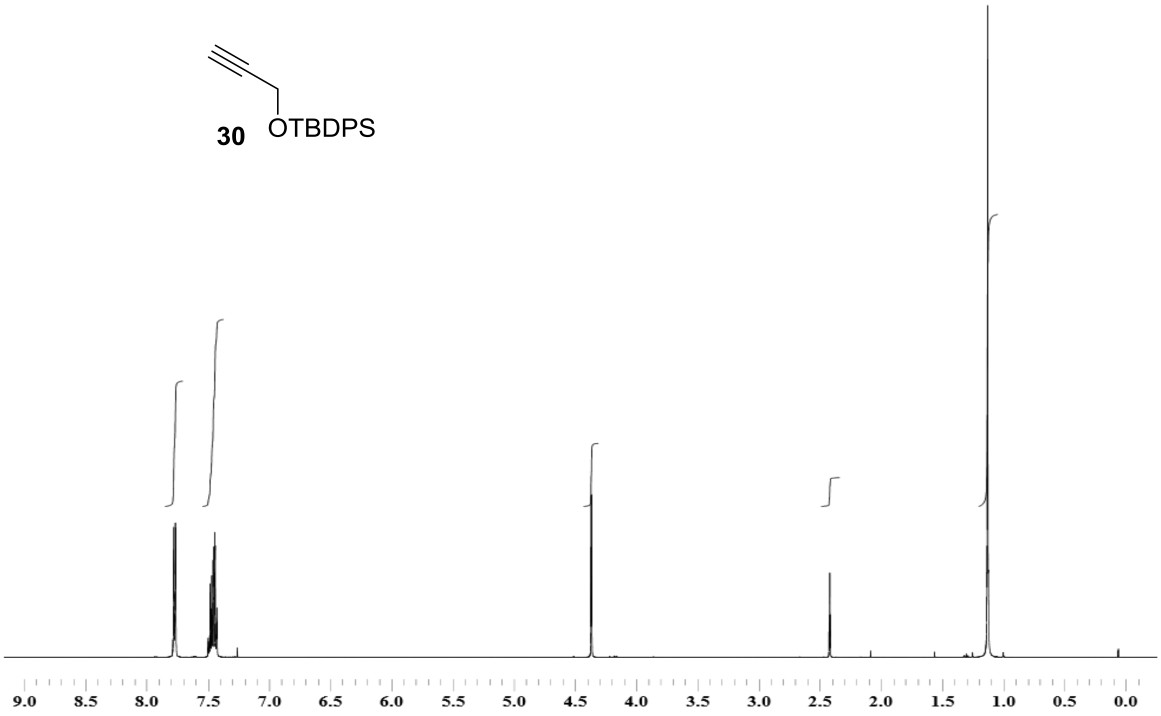

^{1}H NMR Spectrum of compound **30** ($CDCl_3$, 500 MHz)

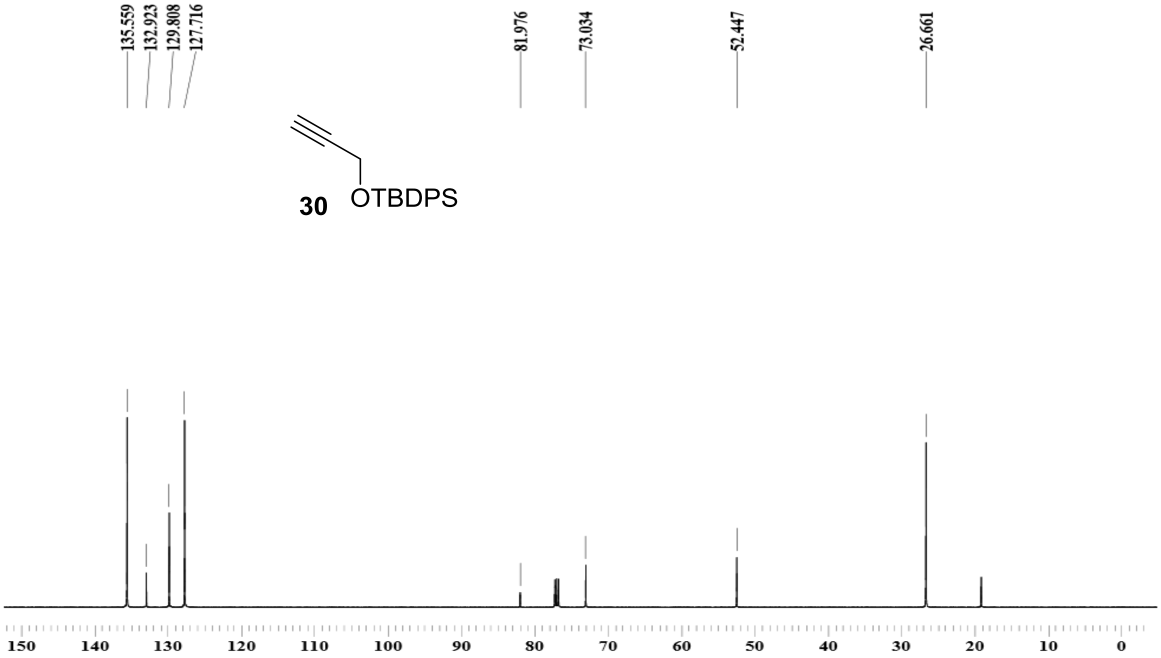

^{13}C NMR Spectrum of compound **30** ($CDCl_3$, 125 MHz)

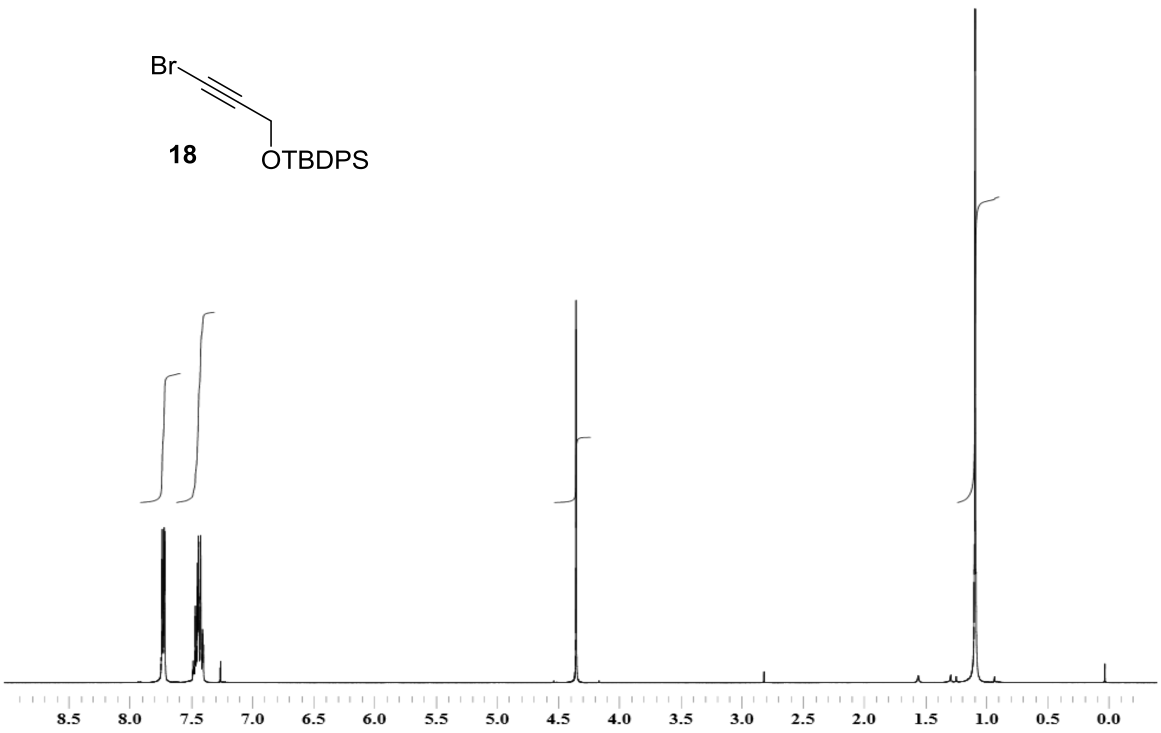

^{1}H NMR Spectrum of compound **18** ($CDCl_3$, 300 MHz)

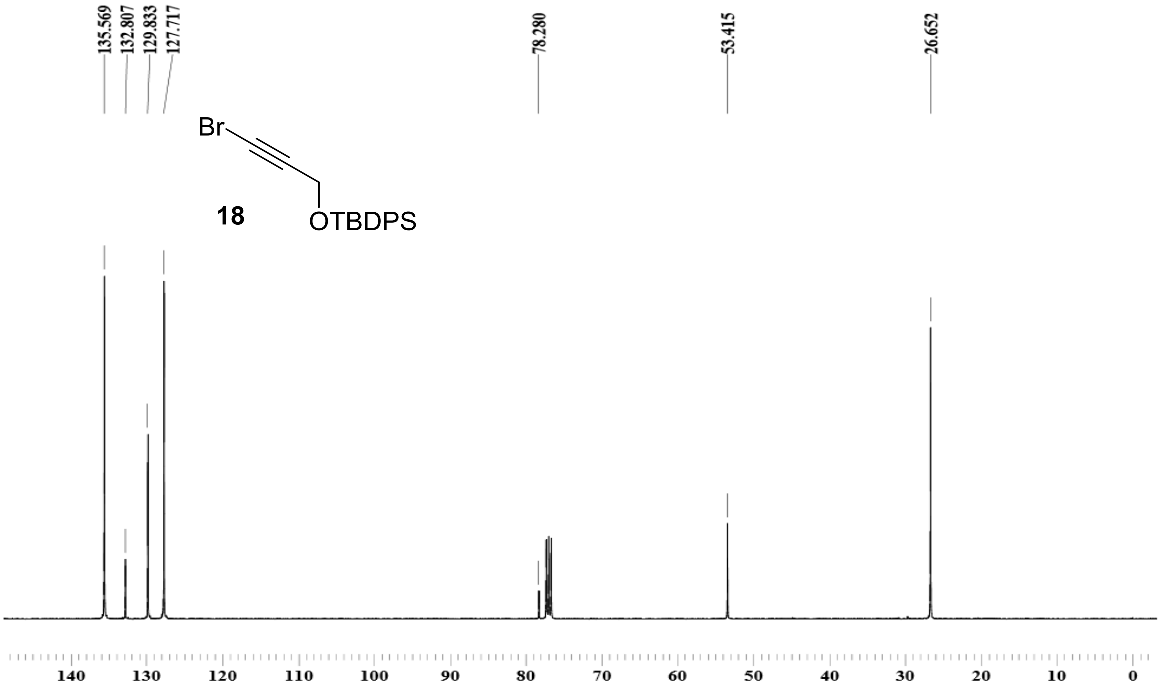

^{13}C NMR Spectrum of compound **18** ($CDCl_3$, 75 MHz)

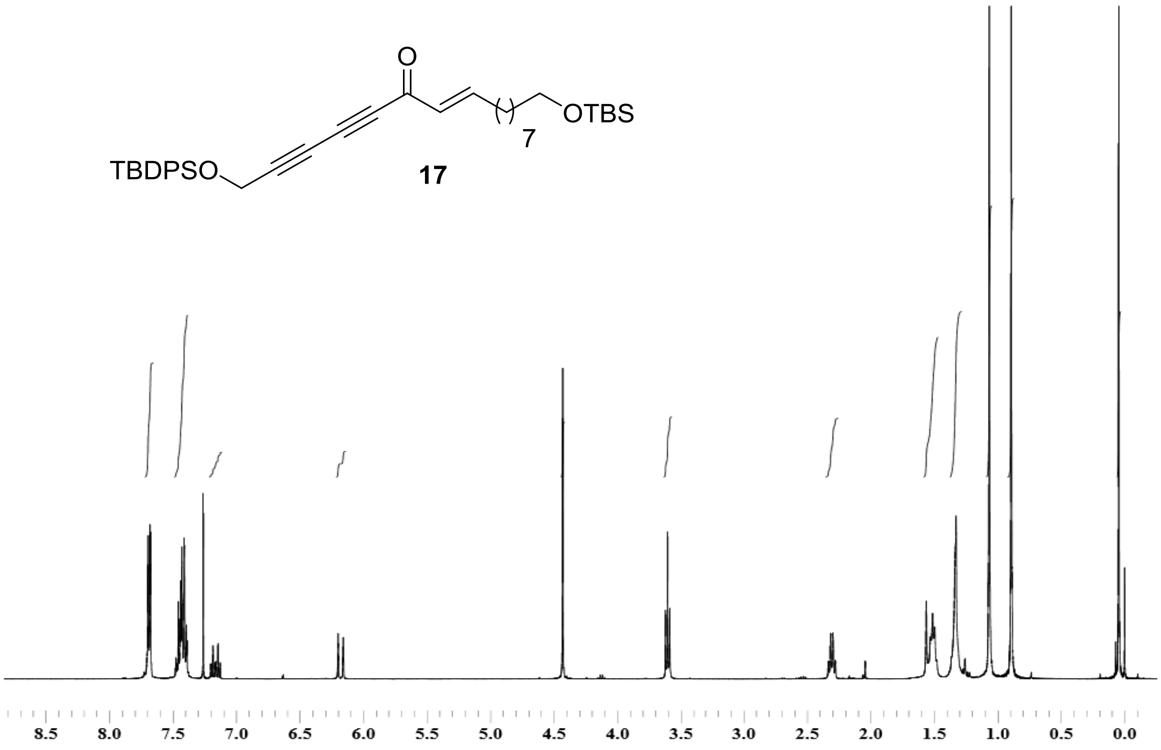

^1H NMR Spectrum of compound **17** ($CDCl_3$, 500 MHz)

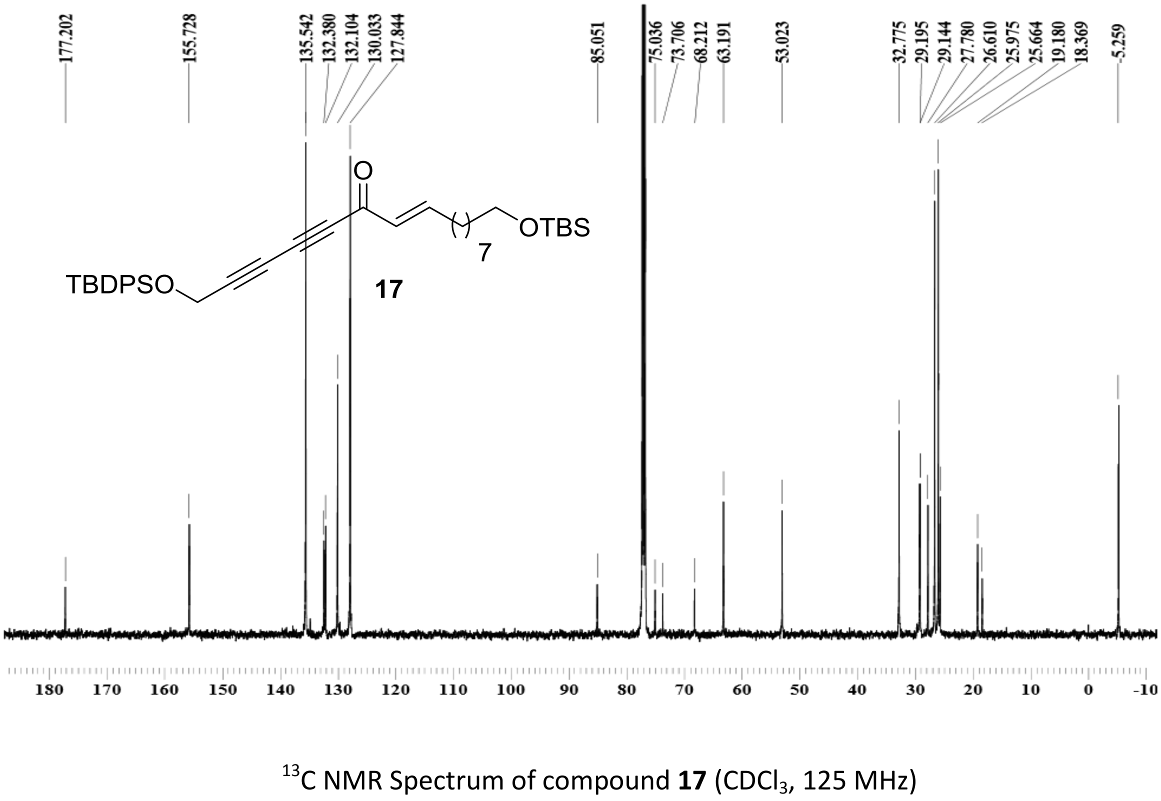

^{13}C NMR Spectrum of compound **17** ($CDCl_3$, 125 MHz)

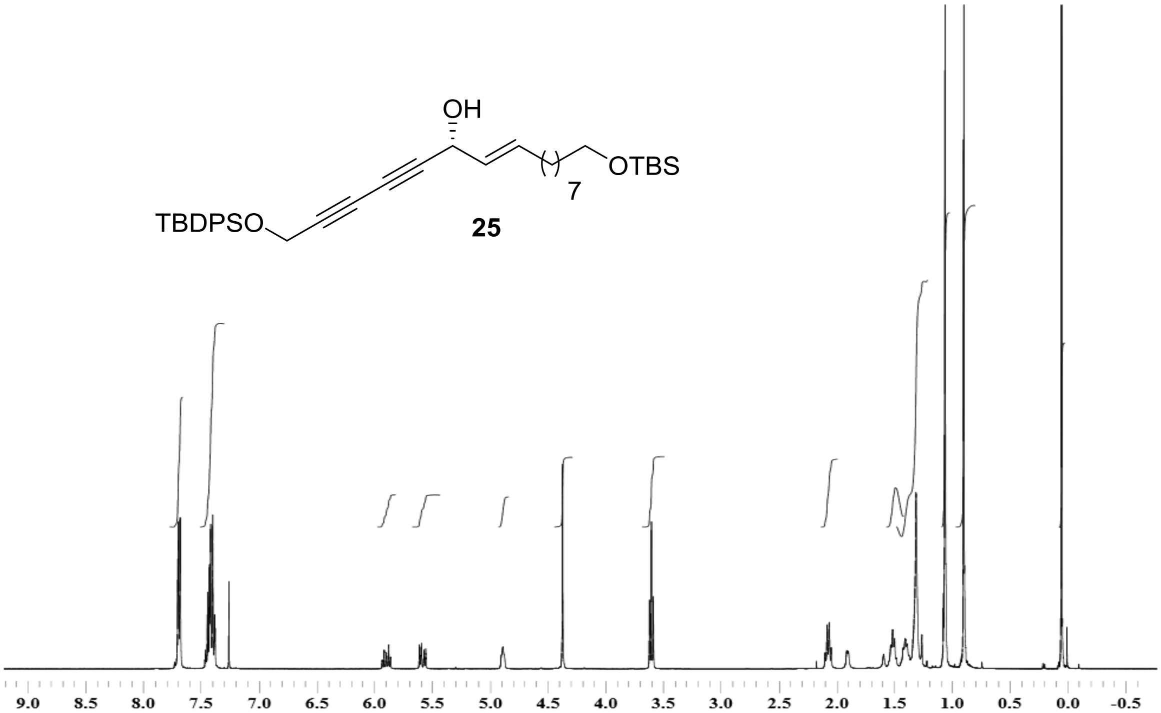

^{1}H NMR Spectrum of compound **25** ($CDCl_3$, 500 MHz)

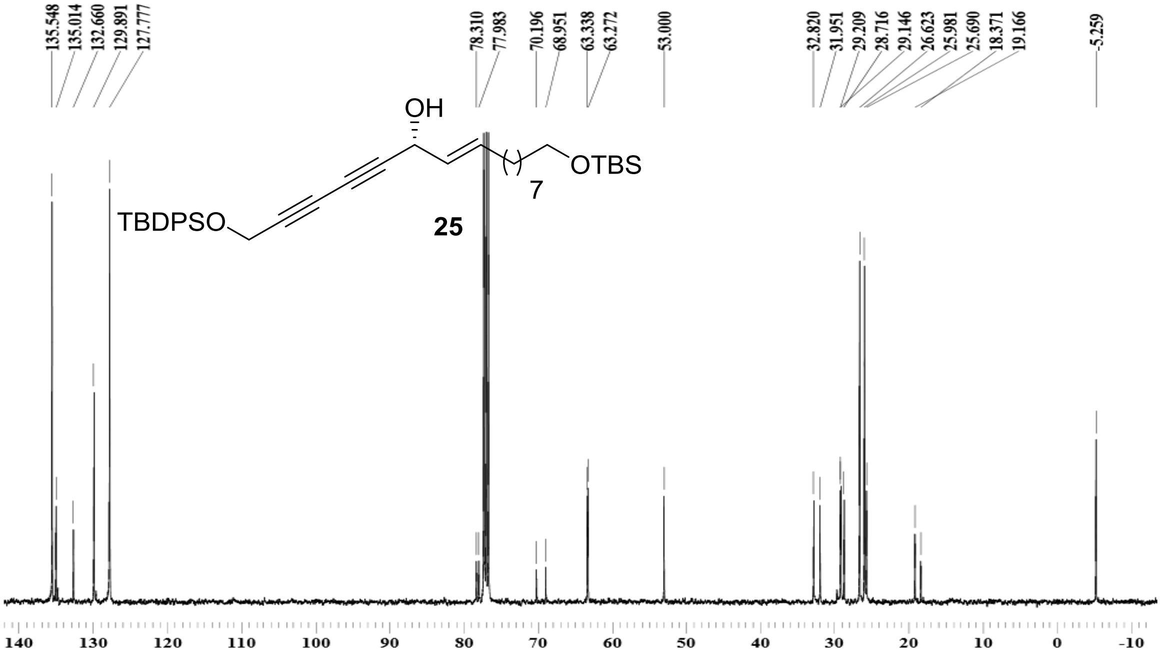

^{13}C NMR Spectrum of compound **25** ($CDCl_3$, 125 MHz)

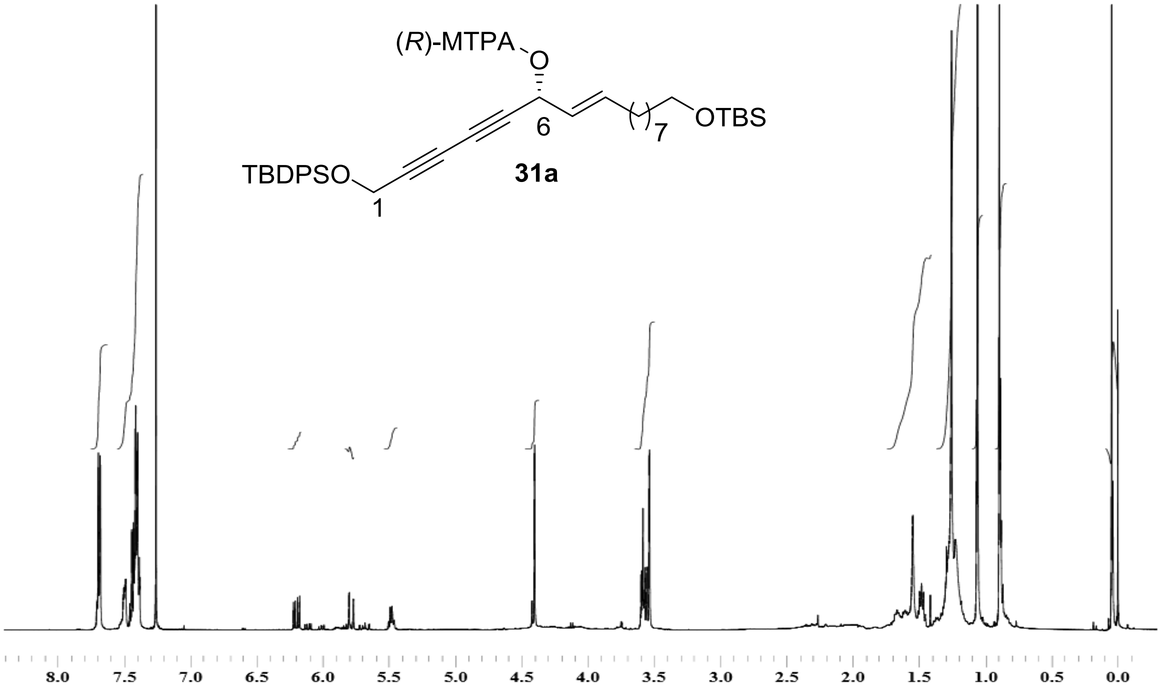

^{1}H NMR Spectrum of compound **31a** ($CDCl_3$, 500 MHz)

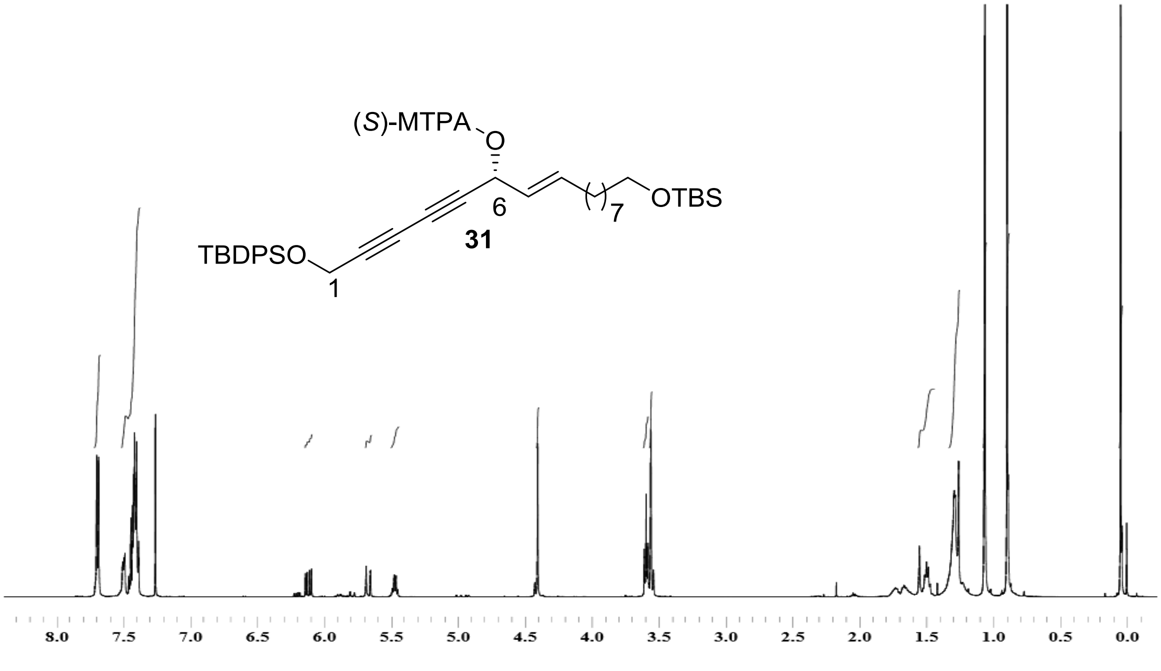

^{1}H NMR Spectrum of compound **31** ($CDCl_3$, 500 MHz)

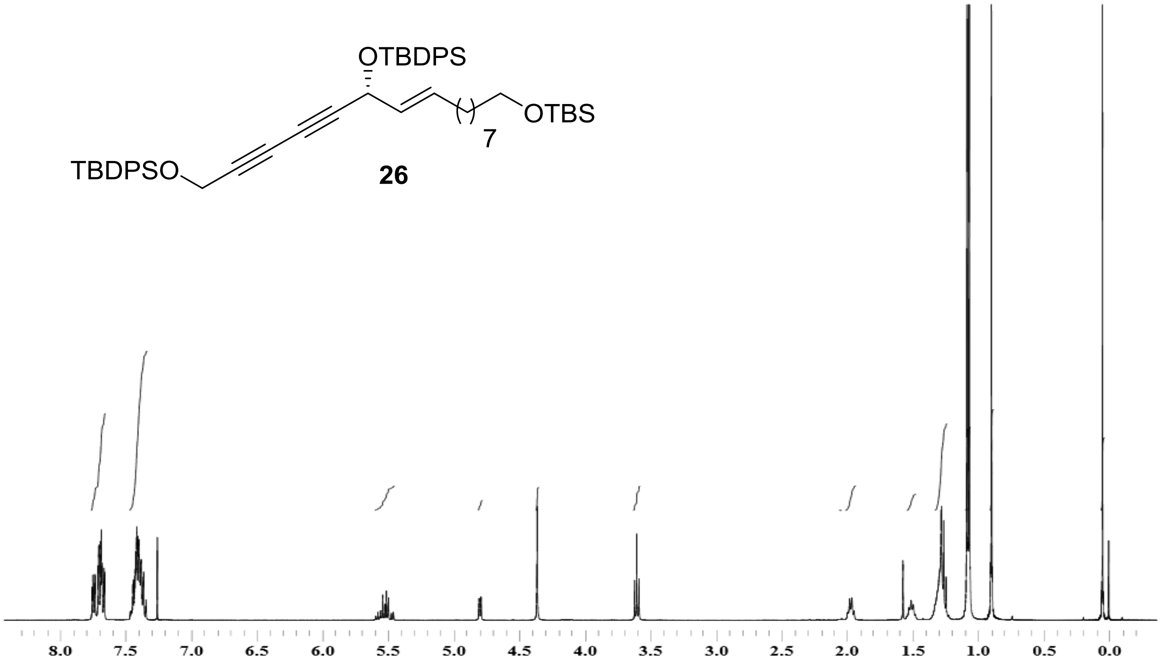

^{1}H NMR Spectrum of compound **26** ($CDCl_3$, 500 MHz)

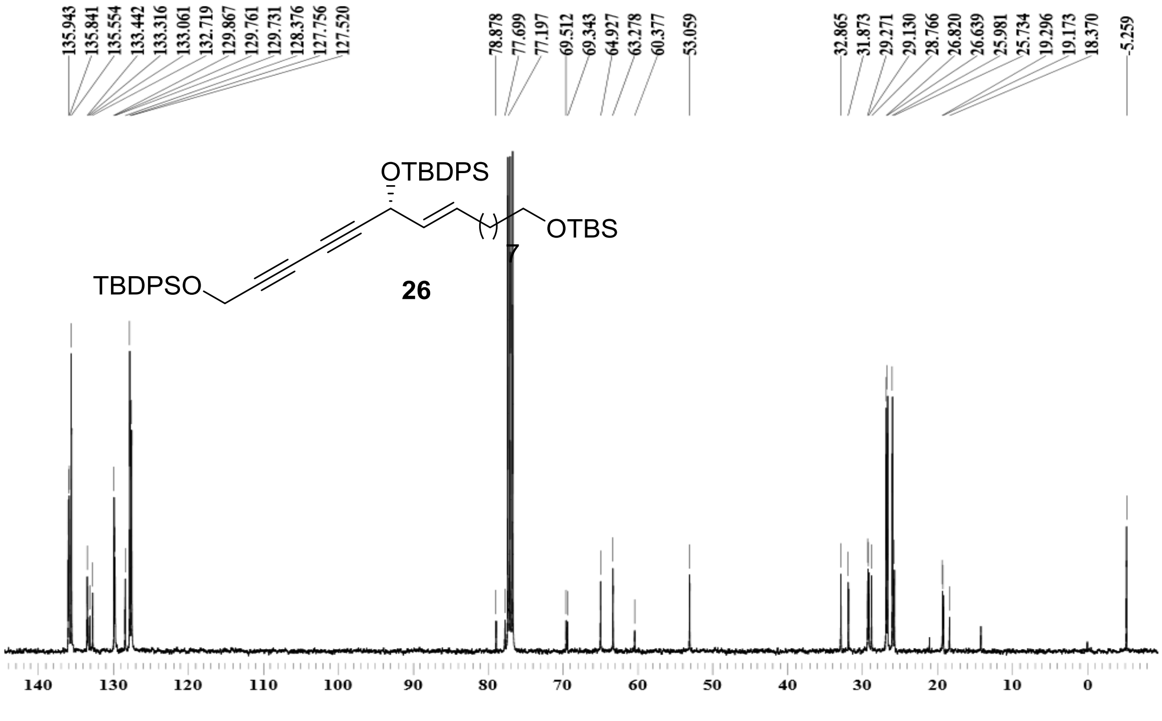

^{13}C NMR Spectrum of compound **26** ($CDCl_3$, 125 MHz)

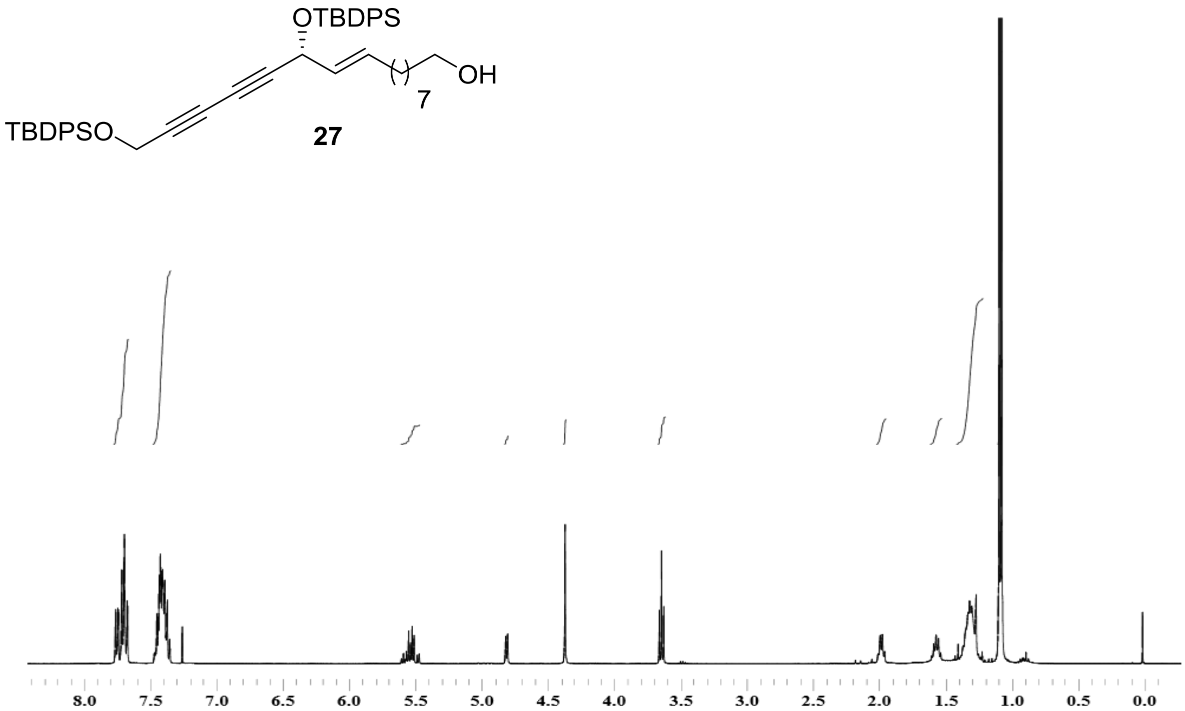

^{1}H NMR Spectrum of compound **27** ($CDCl_3$, 400 MHz)

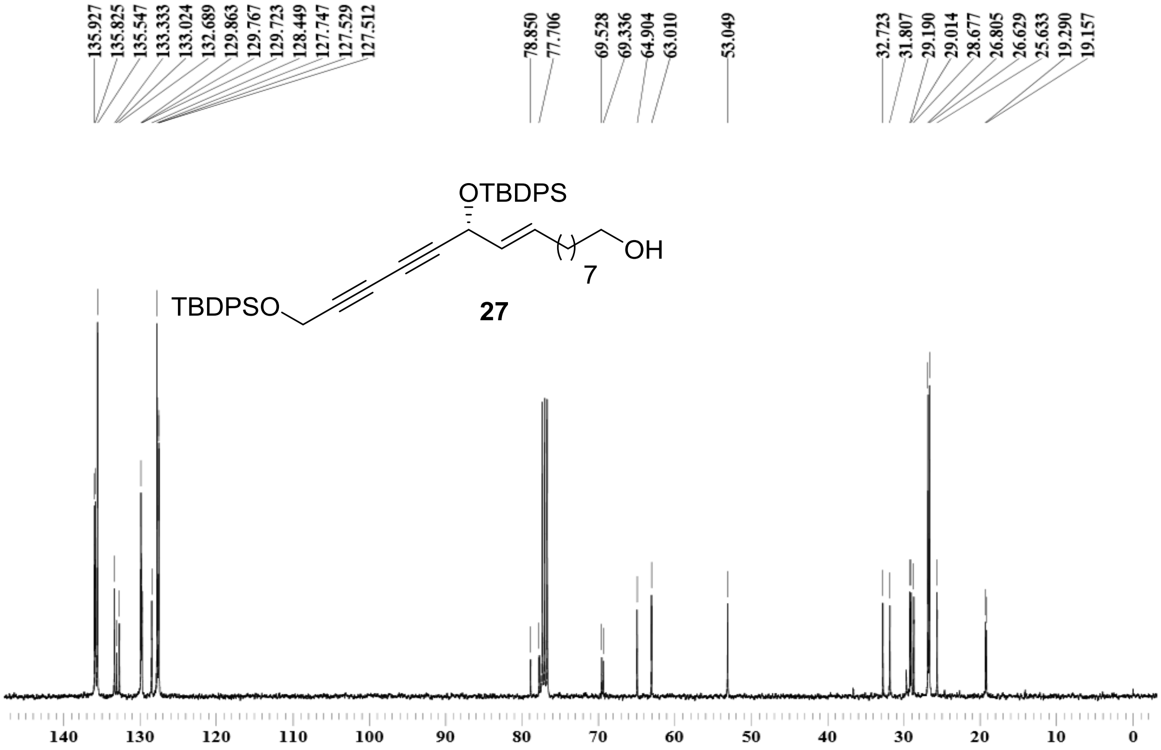

^{13}C NMR Spectrum of compound **27** ($CDCl_3$, 100 MHz)

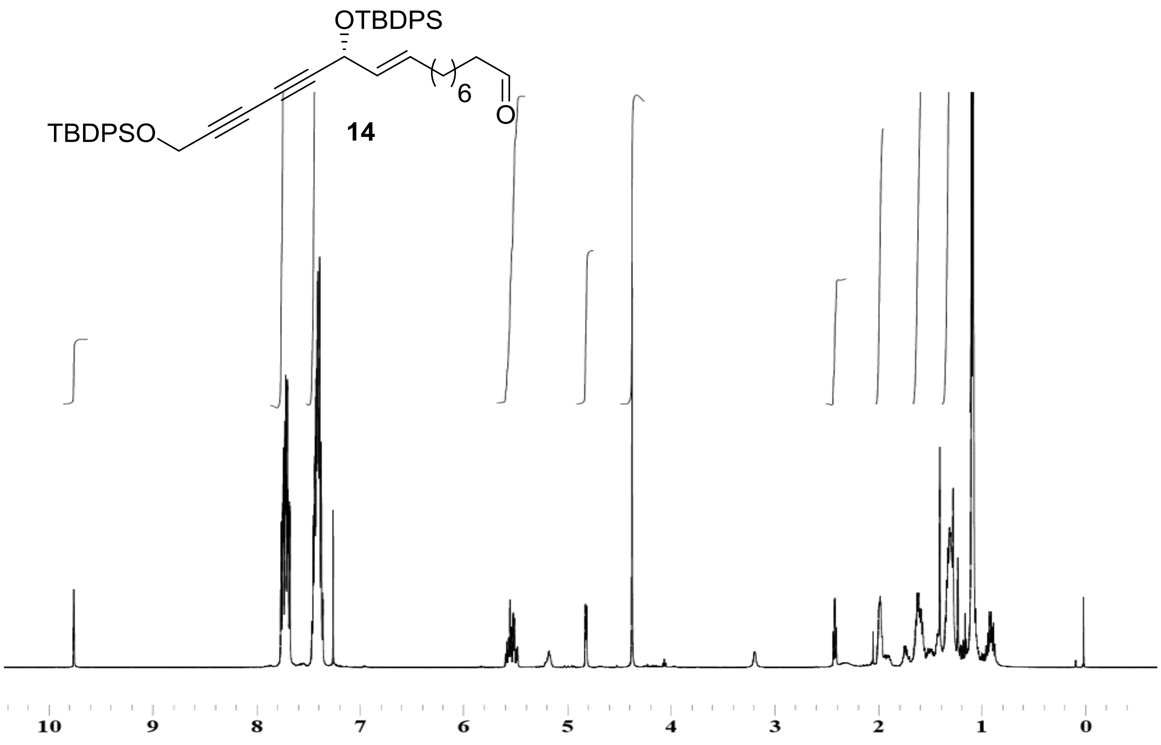

^{1}H NMR Spectrum of compound **14** ($CDCl_3$, 400 MHz)

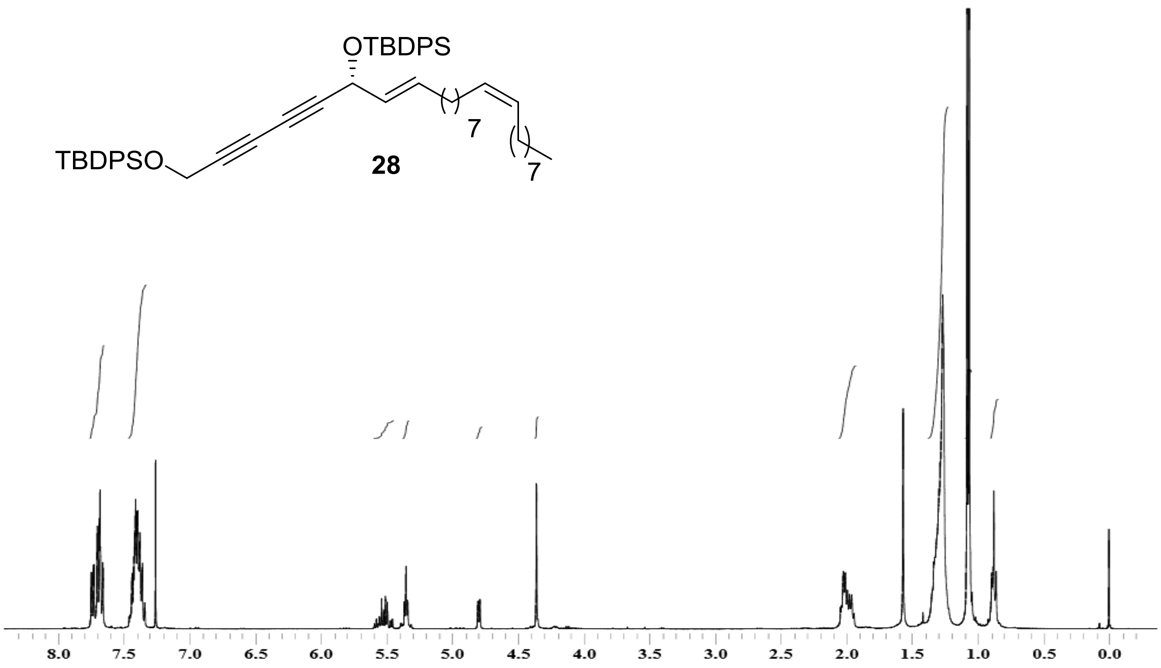

^{1}H NMR Spectrum of compound **28** ($CDCl_3$, 500 MHz)

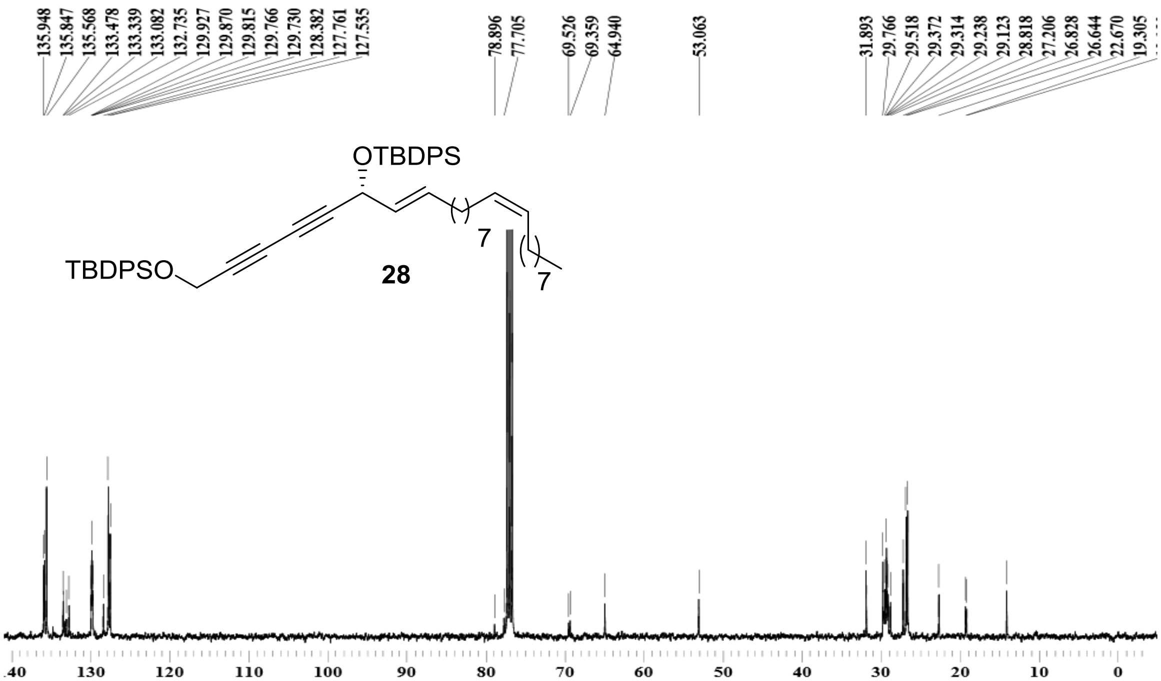

^{13}C NMR Spectrum of compound **28** ($CDCl_3$, 125 MHz)

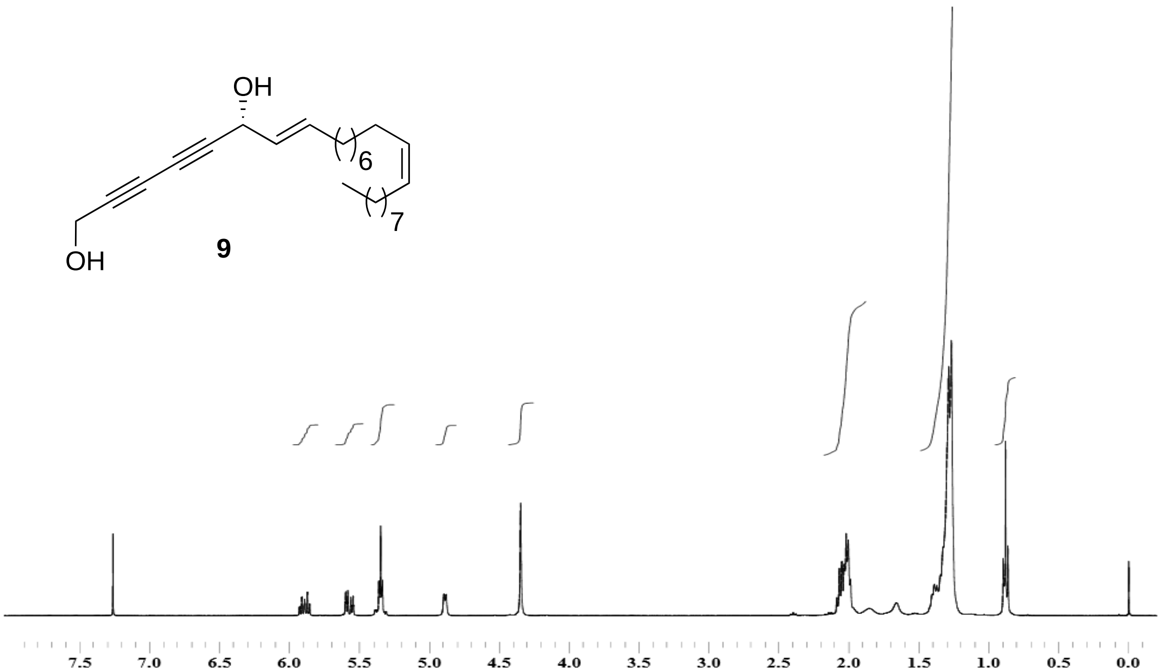

^{1}H NMR Spectrum of compound **9** ($CDCl_3$, 400 MHz)

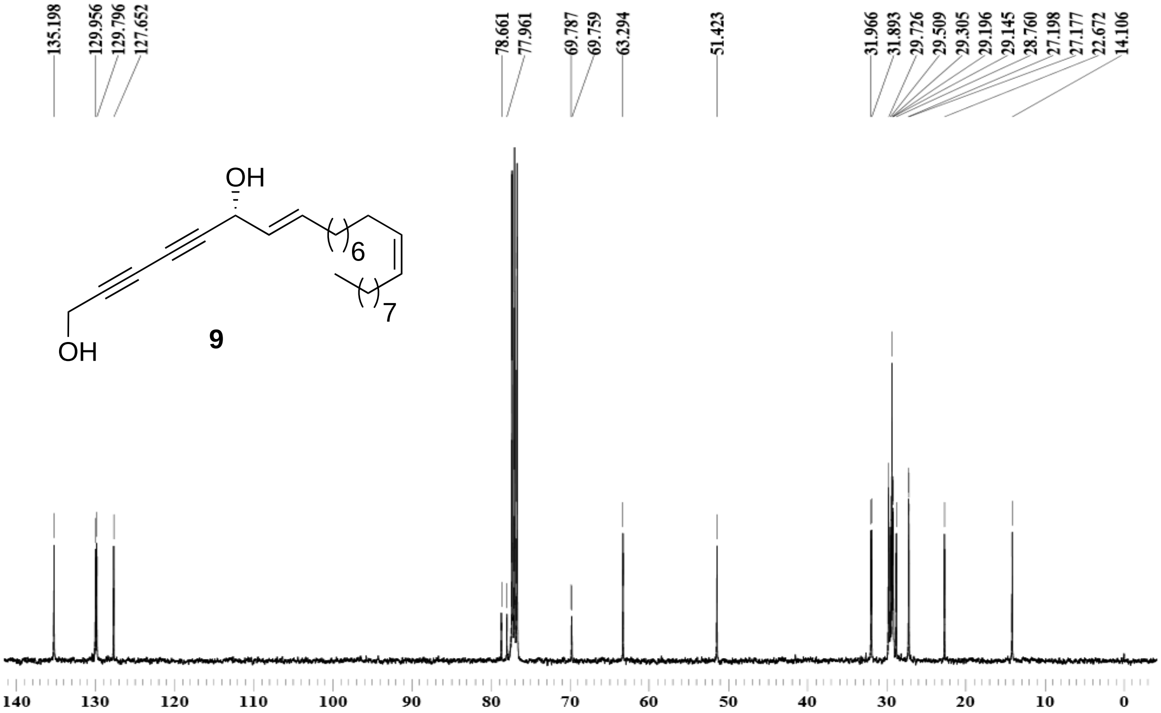

^{13}C NMR Spectrum of compound **9** ($CDCl_3$, 100 MHz)

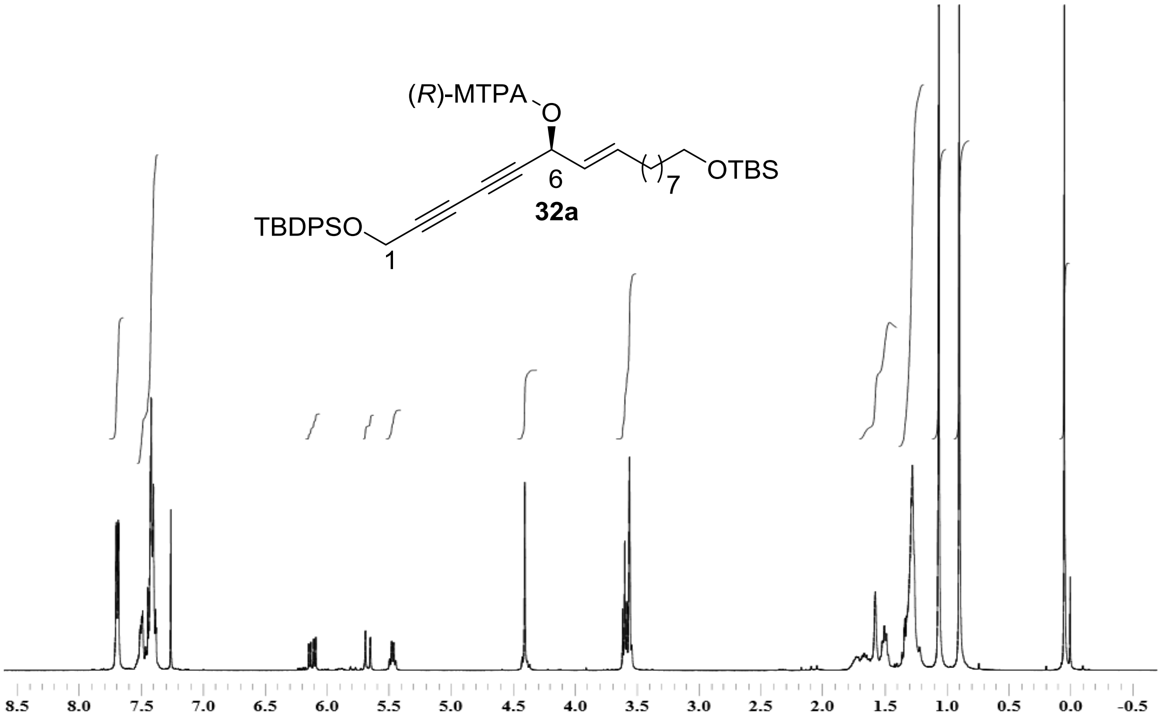

^{1}H NMR Spectrum of compound **32a** ($CDCl_3$, 500 MHz)

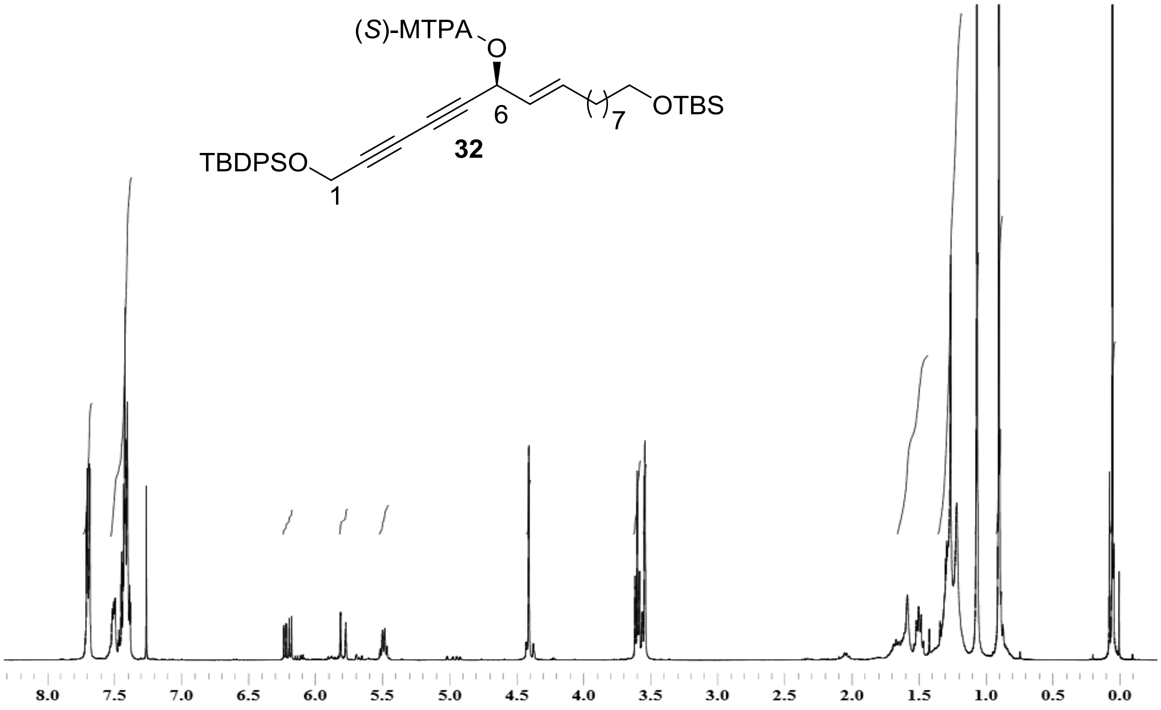

^{1}H NMR Spectrum of compound **32** ($CDCl_3$, 500 MHz)

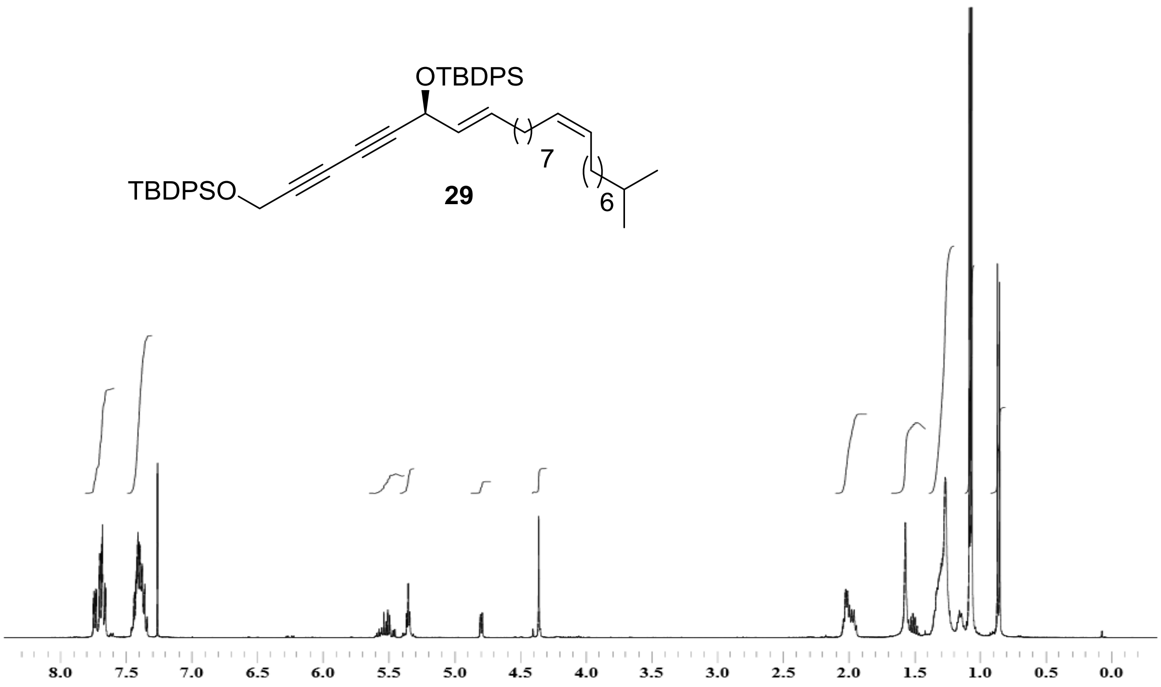

^{1}H NMR Spectrum of compound **29** ($CDCl_3$, 500 MHz)

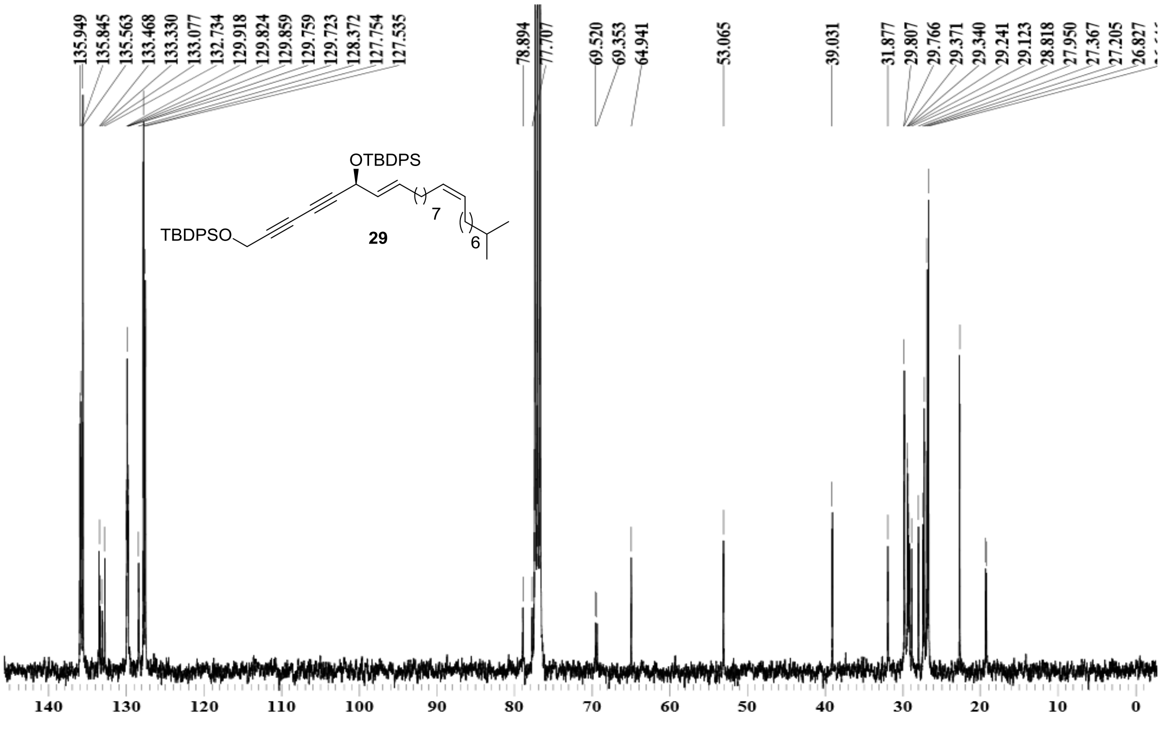

^{13}C NMR Spectrum of compound **29** ($CDCl_3$, 125 MHz)

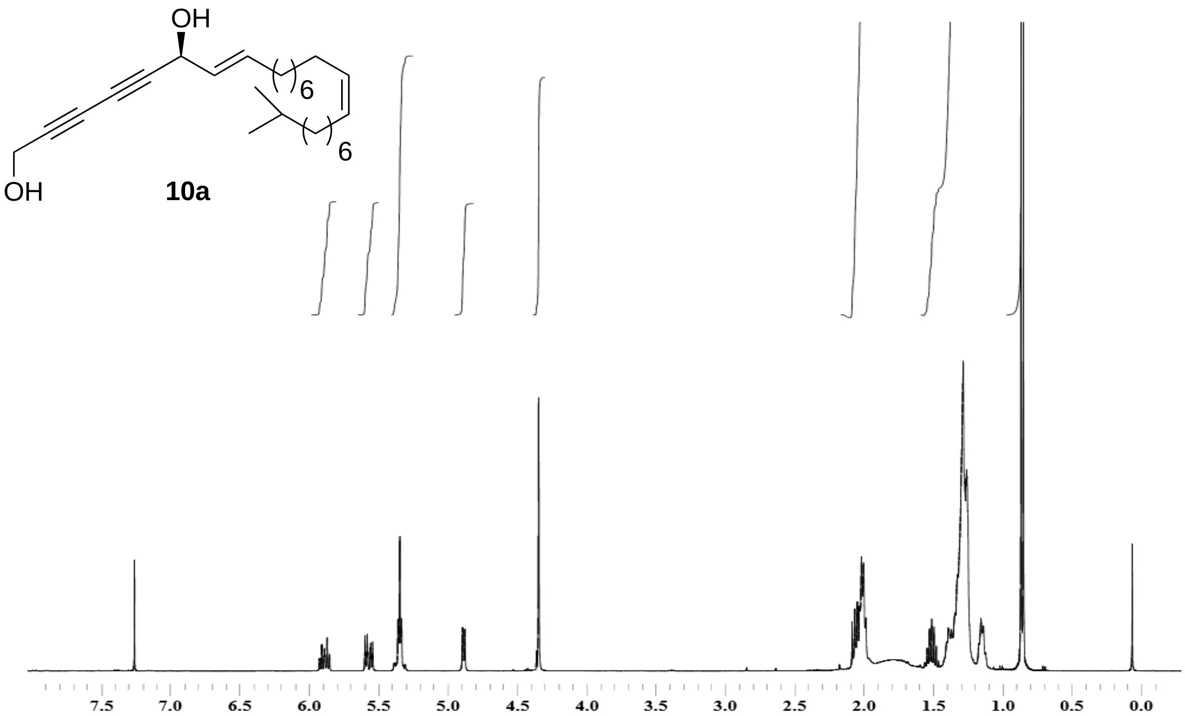

^{1}H NMR Spectrum of compound **10a** ($CDCl_3$, 500 MHz)

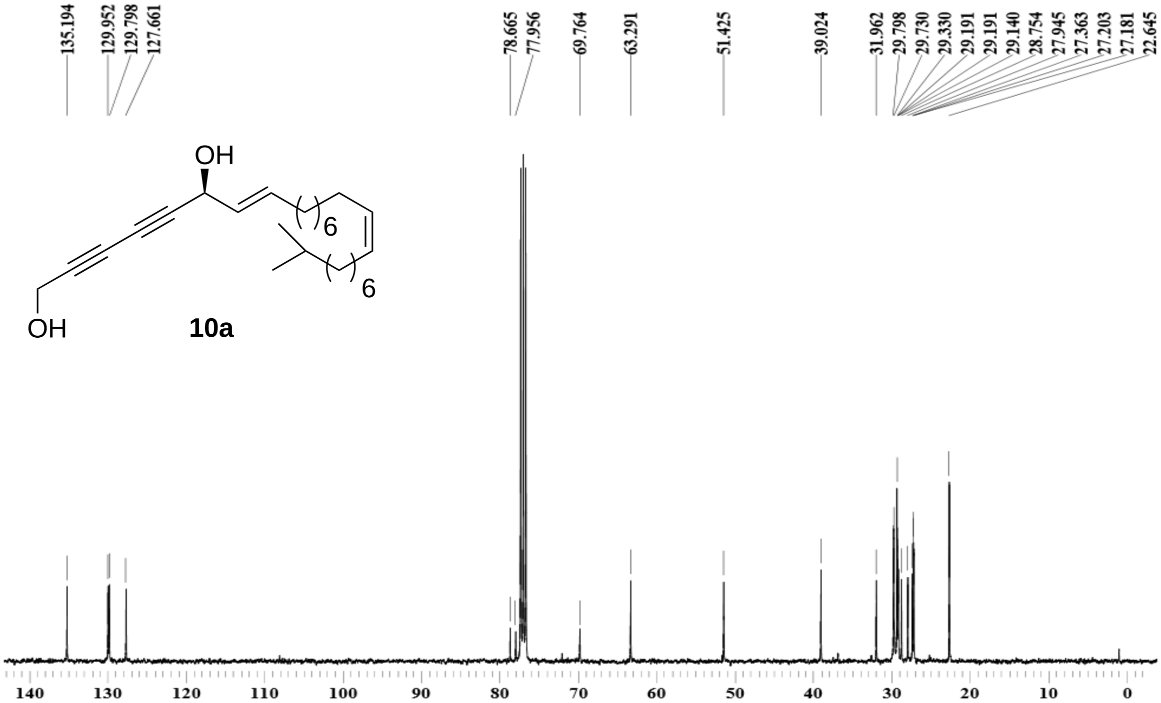

^{13}C NMR Spectrum of compound **10a** ($CDCl_3$, 125 MHz)

LCMS analysis of compound **32a**

IICT-DNPC

LC-MS DATA REPORT

SHIMADZU LabSolutions

Sample Code : PSH-RMOS-832
Data File : 190717.3.lcd
Method : LC-MS -KSB.lcm
Injection Volume : 5
Date Acquired : 7/19/2017 1:11:16 PM
Report File : LC-MS Data Report.lsr
Chromatographic Conditions : Column:KINETEX-F5 (150 X 4.6mm, 5.0u)
Mobile Phase: 90%ACN IN 0.1 F.A
Flow Rate: 1.0 mL/min

Chromatogram

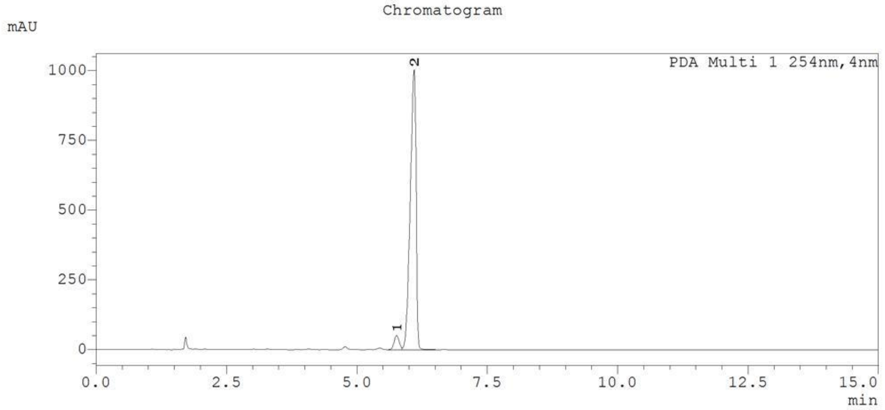

Peak Table

PDA Ch1 254nm

Peak#	Ret. Time	Peak Start	Peak End	Area	Area%
1	5.762	5.589	5.867	349007	4.265
2	6.097	5.867	6.560	7833143	95.735
Total				8182150	100.000

Q1 Scan Positive+
If(SpPrTab==SpPrTab)Spectrum Mode:Averaged 5.662-5.870(1581-1639)
BG Mode:Averaged 0.000-5.160(1-1441)

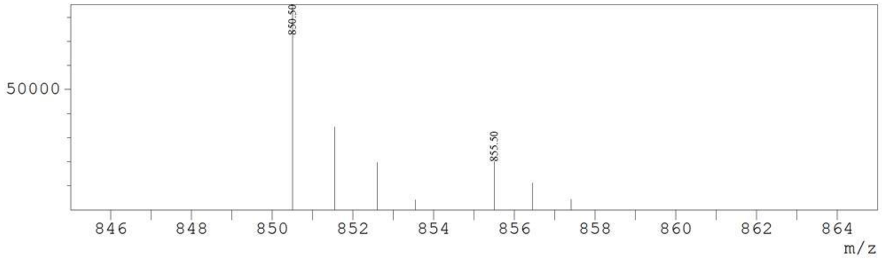

REPORT
